# A soft biosensor with printable responsive hydrogel interfaces for detection and differentiation of blood circulation complications

**DOI:** 10.1093/nsr/nwag058

**Published:** 2026-01-29

**Authors:** Yuqi Qiu, Ganguang Yang, Zhixin Wang, Bo Pang, Sen Zhou, Qingyang Zheng, Tianzhao Bu, Jia Tian, Bing Xue, Junhak Lee, Yeonsik Choi, Zhouping Yin, Changsheng Wu, Yutian Liu, Hao Wu

**Affiliations:** Flexible Electronics Research Center, State Key Laboratory of Intelligent Manufacturing Equipment and Technology, School of Mechanical Science and Engineering, Huazhong University of Science and Technology, Wuhan 430074, China; Flexible Electronics Research Center, State Key Laboratory of Intelligent Manufacturing Equipment and Technology, School of Mechanical Science and Engineering, Huazhong University of Science and Technology, Wuhan 430074, China; Flexible Electronics Research Center, State Key Laboratory of Intelligent Manufacturing Equipment and Technology, School of Mechanical Science and Engineering, Huazhong University of Science and Technology, Wuhan 430074, China; Flexible Electronics Research Center, State Key Laboratory of Intelligent Manufacturing Equipment and Technology, School of Mechanical Science and Engineering, Huazhong University of Science and Technology, Wuhan 430074, China; Flexible Electronics Research Center, State Key Laboratory of Intelligent Manufacturing Equipment and Technology, School of Mechanical Science and Engineering, Huazhong University of Science and Technology, Wuhan 430074, China; Flexible Electronics Research Center, State Key Laboratory of Intelligent Manufacturing Equipment and Technology, School of Mechanical Science and Engineering, Huazhong University of Science and Technology, Wuhan 430074, China; Flexible Electronics Research Center, State Key Laboratory of Intelligent Manufacturing Equipment and Technology, School of Mechanical Science and Engineering, Huazhong University of Science and Technology, Wuhan 430074, China; Department of Hand Surgery, Union Hospital, Tongji Medical College, Huazhong University of Science and Technology, Wuhan 430022, China; Department of Materials Science and Engineering, National University of Singapore, Singapore 117575, Singapore; Department of Materials Science and Engineering, Yonsei University, Seoul 03722, Republic of Korea; Department of Materials Science and Engineering, Yonsei University, Seoul 03722, Republic of Korea; Flexible Electronics Research Center, State Key Laboratory of Intelligent Manufacturing Equipment and Technology, School of Mechanical Science and Engineering, Huazhong University of Science and Technology, Wuhan 430074, China; Department of Materials Science and Engineering, National University of Singapore, Singapore 117575, Singapore; Institute for Health Innovation and Technology, National University of Singapore, Singapore 117599, Singapore; Department of Electrical and Computer Engineering, National University of Singapore, Singapore 119276, Singapore; The N.1 Institute for Health, National University of Singapore, Singapore 117456, Singapore; Department of Hand Surgery, Union Hospital, Tongji Medical College, Huazhong University of Science and Technology, Wuhan 430022, China; Flexible Electronics Research Center, State Key Laboratory of Intelligent Manufacturing Equipment and Technology, School of Mechanical Science and Engineering, Huazhong University of Science and Technology, Wuhan 430074, China; School of Integrated Circuits, Huazhong University of Science and Technology, Wuhan 430074, China

**Keywords:** flexible electronics, soft bioelectronics, blood circulation complications, responsive hydrogel interface, direct printing

## Abstract

Flexible electronics for healthcare applications have been drawing significant attention and show great promise for monitoring blood circulation (e.g. postoperative monitoring of free flaps). However, existing methods for design and fabrication of interfaces with human skin still cannot meet the challenging clinical requirements of superior adhesion during monitoring, and avoiding wound damage during peel-off. Here, we propose a soft biosensor with universal responsive hydrogel interfaces for detecting blood circulation complications. Particularly, we develop thermoresponsive and printable hydrogel inks to rapidly achieve high-precision patterning and wide-range adhesion regulation of interface layers. In clinical cases, the hydrogel biosensor can establish robust hydrogel/flap skin coupling for high-fidelity signal acquisition during monitoring, and ensure benign detachment to prevent tissue injury after monitoring. We achieve precise arterial perfusion monitoring based on the perfusion index (PI) via an 810 nm light source. Additionally, we propose a new metric, the balance index (BI), to monitor venous congestion. By analyzing BI, PI and skin temperature, the biosensor enables accurate detection and differentiation of blood circulation complications. The printable thermoresponsive hydrogel can be adopted as a universal interface in flexible electronics for healthcare applications, and the biosensor represents a promising platform for blood circulation monitoring.

## Teaser

A soft biosensor with printable responsive hydrogel interfaces was developed to detect and differentiate blood circulation complications while ensuring robust coupling and benign detachment with skin.

## INTRODUCTION

In healthcare applications, high-fidelity monitoring of physiological signals plays a crucial role in achieving early disease warning and precise diagnosis [1–3]. However, conventional monitoring systems exhibit high modulus, poor flexibility and low adhesion, rendering it difficult to achieve tight coupling with soft tissues, which severely compromises signal fidelity [4–9]. Taking free-flap monitoring as an example, in clinical practice, free-flap transfer has become the common treatment for soft tissue reconstruction [10]. In the USA, approximately 28 000 cases of free-flap transfer were performed annually in the Department of Plastic Surgery alone [11]. For postoperative free flaps, blood circulation complications such as venous congestion, arterial spasm and arterial occlusion always present a critical challenge to their viability [12–15]. Therefore, timely detection and accurate differentiation of complications are crucial, which enables prompt intervention and targeted clinical management, thereby improving the survival rate of free flaps [16]. However, the most commonly adopted clinical assessments of skin conditions by nurses and surgeons, including the flap color, turgor, temperature

and capillary refill, are subjective and dependent on the caregiver’s experience [17,18]. Other blood circulation monitoring approaches proposed over the past decades (e.g. acoustic Doppler sonography, laser Doppler flowmetry, cuffless blood pressure monitoring etc.) exhibit major disadvantages, including the inability to differentiate between arterial and venous complications, inconvenient data interpretation and high costs (Table S1) [17,19–21]. More recently, several sensors based on optoelectronic techniques including near-infrared spectroscopy (NIRS) and photoplethysmography (PPG) were developed. Nevertheless, conventional NIRS/PPG monitoring systems mainly consist of rigid modules, which severely impede conformal skin/device attachment and potentially impose mechanical stress on the wound site.

To address these issues, developing flexible sensing systems with skin-adhesive properties has emerged as a promising solution [22,23]. These soft systems are fabricated using stretchable materials to enhance mechanical compliance, and seamless integration with skin can be achieved by assembling hydrogel interface layers with superior adhesion, flexibility and biocompatibility [24–27]. Recent progress in laser-patterned conductive polymer hydrogel biosensors has demonstrated enhanced adhesion properties [28–30]. Besides this, incorporating responsive functional components enables the phase transition and tunable performance of interface layers [31]. Notably, for the clinical application of postoperative flap monitoring, due to the inherent fragility of the flap wound, the interface with skin requires not only high but also tunable adhesion to simultaneously ensure: (i) robust coupling with free flap skin for stable signal recording during monitoring; and (ii) gentle detachment from skin to prevent wound tissue damage after monitoring [32,33]. Although self-adhesive hydrogel systems have been reported recently, there are still several limitations including low initial adhesion or the inability to achieve adhesion regulation [34,35]. Additionally, conventional mold-pouring methods face severe limitations in fabricating hydrogel interfaces for on-skin systems, including poor manufacturing precision, time-consuming processes and difficulty in customizable patterning and mold separation [36–38]. Therefore, development of a universal hydrogel interface layer with tunable adhesion and rapid patterning capability to achieve robust device/skin integration and gentle disassembly of flexible blood circulation monitoring systems remains a critical challenge.

In this article, we develop a soft biosensor with printable responsive hydrogel interfaces for accurately detecting and differentiating blood circulation complications. The hydrogel material is designed to serve as the universal interface with human skin/tissues for biosensors in healthcare applications. To achieve high-precision patterning and tunable adhesion of hydrogel interface layers, we develop a printable thermoresponsive *N*-isopropylacrylamide (NIPAM)/zwitterion-based/dopamine (DA)-functionalized hydrogel (NZDH). By incorporating cellulose nanofibers (CNFs) to modulate the rheological properties of hydrogel inks, we achieve high-precision direct printing of hydrogels, completing interface layer patterning of the biosensors within 30 s. Due to the decoration of zwitterionic and catechol groups, the NZDHs demonstrate superior initial adhesion (27.8 kPa). Based on the thermally triggered phase transition mechanism of polyNIPAM (PNIPAM) chains, the hydrogel exhibits wide-range adhesion regulation (>10.8-fold). Specifically, the printed hydrogel biosensor rapidly establishes intimate coupling with skin tissues during postoperative flap monitoring, enabling high-fidelity signal acquisition. After the monitoring concludes, heat-induced temperature elevation of NZDHs leads to rapid adhesion attenuation and benign delamination from flap skin without causing secondary injury. In clinical cases, the hydrogel biosensor collects reflective infrared PPG signals and temperature signals simultaneously to monitor free flaps. The biosensor integrated with an 810 nm light-emitting diode (LED) ensures that the perfusion index (PI) measured from PPG signals correlates exclusively with arterial perfusion, remaining independent of blood oxygenation [39–42]. Meanwhile, based on the finding that venous congestion transforms the standard PPG waveform into a reverse waveform, we propose a new metric, the balance index (BI), to quantitatively detect venous congestion. Animal tests validate the correlation between the BI and PI with different blood circulation states, including venous congestion, arterial spasm (i.e. partial arterial occlusion) and arterial occlusion. Compared with the clinical assessment and a commercial microcirculation monitoring system, the hydrogel biosensor can detect and differentiate blood circulation complications in a timely and accurate manner, with additional advantages of low risk of mechanical damage, low cost and wireless communication. More importantly, the rapidly printed thermoresponsive hydrogel can serve as a universal interface for flexible electronics, effectively enhancing signal fidelity while ensuring benign device detachment, possessing great potential for various healthcare monitoring applications.

## RESULTS AND DISCUSSION

### Design and overview of the hydrogel biosensor

**Figure 1.**
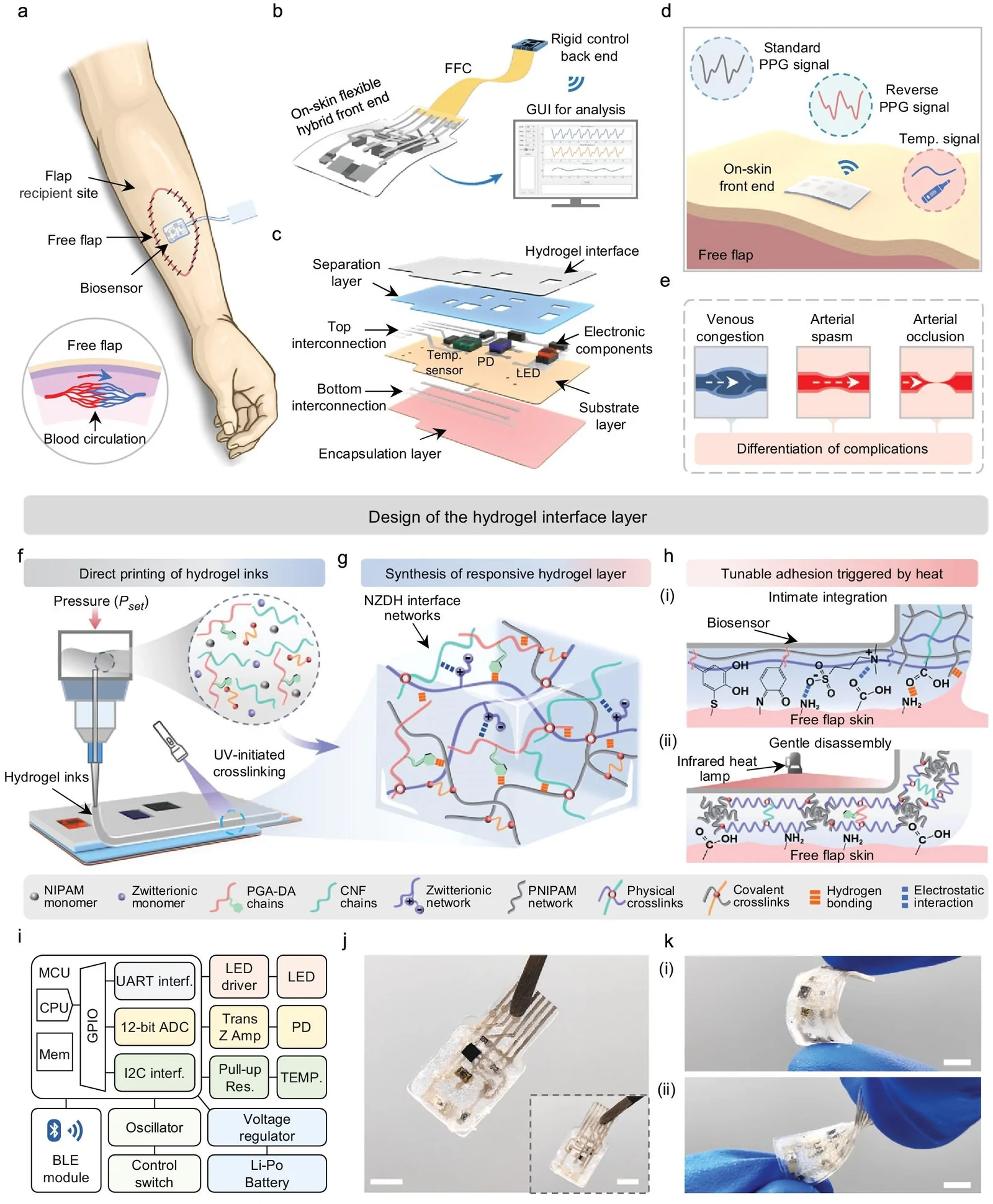
Design and architecture of the hydrogel biosensor. (a) Schematic diagram of the biosensor applied to the flap recipient site to monitor blood circulation. (b) Schematic overall structure of the biosensor. FFC, flexible flat cable; GUI, graphical user interface. (c) An exploded 3D model of the layer assembly of the front end. (d) Illustration of the front end of the biosensor attached to the free flap conformally to detect the standard PPG signal, reverse PPG signal and temperature signal. Temp., temperature. (e) Schematic of blood circulation complications of postoperative free flaps, including venous congestion, arterial spasm and arterial occlusion. (f) Schematic of the direct printing process for hydrogel inks. (g) Compositions of the crosslinked hydrogel networks initiated via UV light. (h) Adhesion regulation mechanisms of hydrogel interfaces, enabling intimate integration with free flaps (i) and gentle disassembly triggered by an infrared heat lamp (ii). (i) Block diagram of the electronic system of the biosensor. MCU, microcontroller unit; Mem, memory; GPIO, general-purpose input/output port; UART, universal asynchronous receiver/transmitter; ADC, analog-to-digital converter; I2C, inter-integrated circuit; interf., interface; Trans Z Amp, trans-impedance amplifier; Res., resistors; TEMP., temperature sensor; Li-Po, lithium polymer. (j) Photographs of the front end of the biosensor. The inset shows the back side of the front end. (k) Photographs of the hydrogel biosensor during bending (i) and twisting (ii). Scale bars, 5 mm (j and k).

Figure 1a shows the schematic illustration of the hydrogel biosensor attached to a free flap to monitor blood circulation (inset). The biosensor is constructed based on the assembly of an on-skin flexible hybrid front end, a rigid control back end and a flexible flat cable (FFC), where the front end achieves signal acquisition, the back end manages system control, signal processing and wireless data transmission, and the FFC implements interconnections (Fig. 1b). All signals collected can be wirelessly transmitted to a laptop via Bluetooth Low Energy (BLE) technology for real-time display and medical analysis within a graphical user interface (GUI). The key component of the biosensor is the on-skin front end, which is prepared through flexible hybrid fabrication processes (Fig. S1). Figure 1c illustrates the exploded view of the front end that highlights seven key layers. Polydimethylsiloxane (PDMS) is employed as the substrate layer, the encapsulation layer and the separation layer. Electrically conductive composites (ECCs), which have high conductivity and stretchability, are utilized for preparing flexible conductive layers and bonding electronic components [43]. It is noted that the front end integrates an LED emitting at 810 nm, a sensitive photodiode (PD), a temperature sensor and other passive electronic components including resistors and capacitors to collect PPG and temperature signals (Fig. 1d). Particularly, we identified a type of abnormal PPG signal from free flaps experiencing venous congestion, and defined it as the reverse PPG signal to distinguish its morphological difference from the standard PPG signal. In clinical scenarios, analysis of standard PPG signals, reverse PPG signals and temperature signals enables accurate identification of different blood circulation complications, including venous congestion, arterial spasm and artery occlusion (Fig. 1e).

To achieve tight coupling and benign detachment with flap skin, we print a thermoresponsive hydrogel interface layer (i.e. NZDH) onto the biosensor. As shown in Fig. 1f, we first prepare hydrogel prepolymer solutions by mixing functional monomers, then formulate printable hydrogel inks by adjusting viscosity through controlled additions of CNFs. These inks are loaded into nozzles and printed onto flexible substrates under the optimized pressure and speed. Meanwhile, we synthesize the thermally responsive NZDH interface layer via UV-induced crosslinking. Figure 1g depicts the hydrogel compositions, consisting of PNIPAM, zwitterionic chains [i.e. poly(3-dimethyl(methacryloyloxyethyl) ammonium propane sulfonate) (PDMAPS)], CNF, and polyglutamic acid grafted with DA (PGA–DA) through physical entanglement and chemical crosslinking. The thermally triggered adhesion regulation mechanism of NZDHs operates as follows: at room temperature (RT), the PNIPAM network maintains extended chain conformations through hydrogen bonds between hydrophilic amide groups and water molecules, dramatically enhancing the energy dissipation of hydrogels. Combined with synergistic effects among catechol groups, carboxyl and zwitterions, NZDHs achieve intimate integration with skin via Michael addition, Schiff base formation, electrostatic interactions and hydrogen bonding, enabling high-quality signal acquisition [Fig. 1h(i)]. It is noted that the infrared heat lamps are commonly used to facilitate flap rehabilitation in clinical wards. Therefore, after the monitoring is completed, the temperature of hydrogel interfaces is conveniently increased via a lamp. The hydrogen bonds between PNIPAM chains and water molecules are disrupted, triggering the transition of hydrated PNIPAM chains into hydrophobic cluster aggregates. This transition increases network entanglement and crosslinking density, substantially reducing the adhesion and energy dissipation capacity of hydrogels, thereby facilitating gentle detachment of hydrogel/skin interfaces without causing secondary tissue damage [Fig. 1h(ii)]. Figure 1i presents a functional diagram of the electronic system. Figure 1j and Fig. S2 show the photographs of the biosensor. It is highlighted that the hydrogel biosensor demonstrates superior flexibility, which is capable of withstanding both bending and twisting deformations (Fig. 1k).

### Direct-printing patterning of hydrogel interface materials

**Figure 2.**
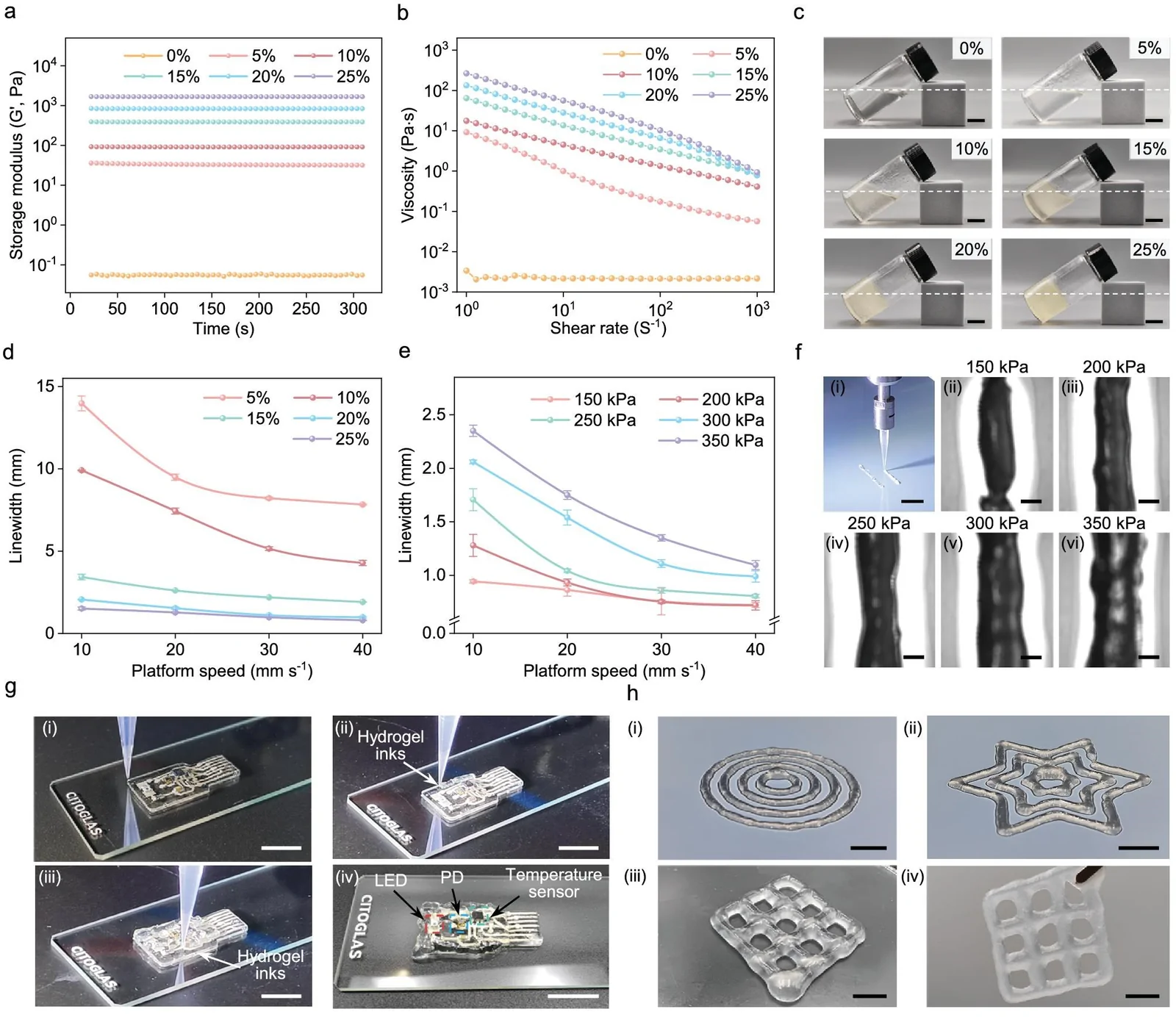
Synthesis and characterizations of the directly printable hydrogel inks. (a) Storage modulus (G′) changes over time of hydrogel inks with different weight ratios of CNF/NIPAM. (b) Viscosity–shear rate curves of corresponding hydrogel inks. (c) Photographs of hydrogel inks with CNF/NIPAM at weight ratios of 0%, 5%, 10%, 15%, 20% and 25%. White dashed lines highlight the rheological differences among the inks. (d) The influence of platform speed on the printed linewidth of different hydrogel inks. (e and f) Printed linewidth of hydrogel inks versus platform speeds under different pressures (e) and corresponding microscope images (f). (g) Printing process of hydrogel inks to fabricate the interface layer of biosensors. (h) Printed 2D and 3D structures via hydrogel inks. Data in (d) and (e) are presented as mean ± SD; *n* = 3. Scale bars, 10 mm (c, g, h), 20 mm (f(i)) and 500 μm (f(ii–vi)).

Flexible electronic systems typically require customizable patterning of interface layers. Specifically, certain areas of integrated electronics or functional sensors require direct contact with skin for accurate data collection, while other areas need to be covered by adhesive interface materials to maintain contact with skin. Currently, the widely adopted mold-pouring methods for hydrogel interfaces have limitations, including the incapability of achieving customized patterning, poor manufacturing precision and time-consuming processes [36–38]. To develop a universal hydrogel interface fabrication approach for flexible electronics, we propose a direct-printing strategy of the hydrogel materials for high-precision and rapid patterning by synthesizing printable inks (Fig. S3). Firstly, we adjusted the viscosity of hydrogel precursor solutions by modifying the CNF chains and conducted rheological property tests on pre-crosslinked NZDH solutions with different weight ratios of CNF/NIPAM. As shown in Fig. 2a, b and Fig. S4, as the weight ratio of CNF/NIPAM increases, the storage modulus (G′), loss modulus (G′′), and viscosity of hydrogel inks also show a continuous increase trend. Compared with the NZDH precursor solutions without CNF/NIPAM (0.06 Pa, 0.02 Pa, and 0.003 Pa·s), the hydrogel solution with CNF/NIPAM at a weight ratio of 25% exhibits dramatically higher initial storage modulus (G′, 1684 Pa), loss modulus (G′′, 228.6 Pa) and viscosity (264.1 Pa·s). This is attributed to the formation of dense hydrogen bonds between the CNF chains and pre-crosslinked hydrogels, resulting in more compact physical entanglement. In the three-interval thixotropy test (3ITT), hydrogel precursor networks modified with CNFs demonstrate excellent shape-retention capabilities, which is beneficial for constructing stable skin/hydrogel interfaces (Fig. S4c). As shown in Fig. 2c, the tilting experiments also show that the hydrogel inks gradually become more viscous with the increasing weight ratio of CNF/NIPAM.

To quantitatively evaluate the influence of different parameters (such as ink viscosity, pressure and platform speed) on the printing performance of hydrogel inks, we recorded the corresponding printing trajectories of inks with different weight ratios of CNF/NIPAM. As shown in Fig. 2d, Figs S5 and S6, and Movie S1, as the weight ratio of CNF/NIPAM in the hydrogel inks increases from 5% to 25% with a platform speed of 10, 20, 30 and 40 mm/s, the linewidth of the ink printing trajectory shows a continuously decreasing trend. Notably, at a lower CNF/NIPAM weight ratio (<10%), the ink presents excessive fluidity, leading to diffusion during deposition. Moreover, the inks with a weight ratio of 15%–20% are printable, while a higher weight ratio (>25%) is prone to cause nozzle clogging due to the increased viscosity of inks. We further captured the printing trajectories of hydrogel inks with different extrusion pressures and platform speeds (Fig. 2e and f, Fig. S7). It is noted that when the pressure is 150 kPa with a platform speed from 10 to 40 mm/s, the linewidth of inks decreases from 0.94 to 0.72 mm. Considering both the printing accuracy and efficiency of hydrogel inks, we determined the optimal printing parameters: a CNF/NIPAM weight ratio of 20%, platform speed of 40 mm/s and printing pressure of 300 kPa.

We also demonstrate the patterned printing of hydrogel interface layers for the fabrication of the hydrogel biosensor (Fig. 2g). The hydrogel inks are directly printed on PDMS substrate, which can be finished within 30 s with a linewidth precision below 720 µm (Movie S2). Specifically, regions designated for the LED, PD and temperature sensor in the soft device are not printed with hydrogel inks, thereby preserving precise locations for direct contact with skin for high-fidelity signal acquisition. Meanwhile, the remaining areas of the biosensor are patterned with hydrogel interface layers for tunable adhesion. Notably, the hydrogel inks can sustain 3D structural integrity without requiring supporting structures/molds during the printing process. The thermoresponsive hydrogel networks can be obtained afterwards by direct photo-polymerization via UV irradiation. By adopting the direct-printing strategy proposed in this work, the issues presented by the conventional mold-pouring of hydrogels can be effectively overcome. To validate the printing universality of hydrogel inks, we also print 2D patterns and devices (such as rings, hexagrams, hydrogel electrodes and hydrogel capacitive sensors) and 3D models (such as grids) (Fig. 2h, Fig. S8, Movie S3). We further conducted a comparative analysis between hydrogel layers fabricated via direct-printing methods in this work and existing hydrogel interfaces for bioelectronics (Table S2). It is noteworthy that the hydrogel interface materials in this work exhibit outstanding performance including direct printability and rapidly high-resolution patterning, dramatically improving manufacturing accuracy and efficiency for hydrogel electronic systems.

### Characterizations of responsive hydrogel layers with tunable adhesion

**Figure 3.**
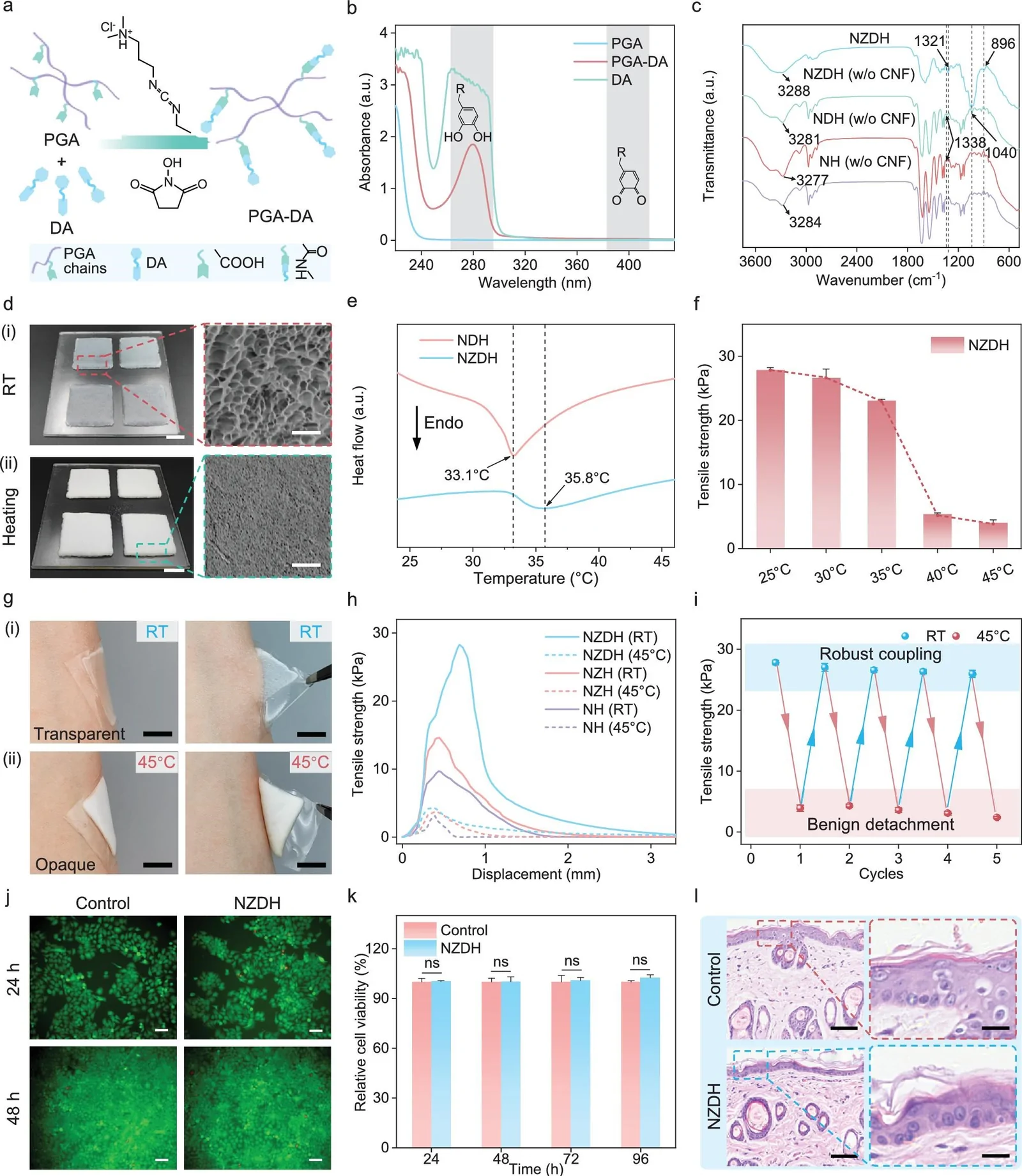
Thermoresponsive and biocompatible properties of hydrogel interface layers. (a) Schematic illustration of the synthesis of PGA–DA. (b) UV–vis spectra of PGA, PGA–DA and DA. (c) FTIR-ATR spectra of different thermoresponsive hydrogels. (d) Photographs of printed hydrogels under RT/heating conditions (left) and corresponding SEM images showing hydrogel network structural changes (right). (e) Comparisons of temperature–heat flow curves of NDHs and NZDHs. Endo down: the downward peak represents an endothermic peak. (f) Adhesion strength of NZDHs when affixed to the pigskin at various temperatures. (g) Demonstration of robust coupling (RT) and facile detachment (45°C) between hydrogel layers and skin. (h) Adhesion curves of thermoresponsive hydrogels with different compositions at 45°C and RT, respectively. (i) Switchable adhesion of NZDHs on the pigskin during RT–45°C cycles. (j) Fluorescence micrographs of HaCaT cells cultured with the NZDH and control groups, stained with Calcein-AM (viable cells, green) and propidium iodide (dead cells, red). (k) Relative cell viability of HaCaTs cultured in NZDH-incubated high-glucose Dulbecco’s modified Eagle medium (HG-DMEM) calculated via CCK-8 assay. (l) Hematoxylin and eosin (H&E)-stained histological sections of skin samples (left) with magnified views (right). *P* values are determined by two-sided Student’s *t*-test between two groups and ANOVA with Tukey’s *post hoc* test between multiple groups, respectively; *P < 0.05, **P < 0.01 and ***P < 0.001; ns, not significant. Data in (f), (i) and (k) are presented as mean ± SD; *n* = 3. Scale bars, 10 mm (d(left) and g), 20 μm (d(right)), 100 μm (j, l(left)) and 25 μm (l(right)).

To fabricate the thermoresponsive hydrogel interface layer (i.e. NZDH), we first improved the initial adhesion of hydrogels by modifying networks with adhesive functional components such as PGA–DA and zwitterionic chains (i.e. PDMAPS). To verify the successful grafting of catechol groups onto the PGA chains, we characterized the synthesized PGA–DA using ultraviolet–visible spectroscopy (UV–vis) and proton nuclear magnetic resonance (^1^H NMR) (Fig. 3a, Note S1). As shown in Fig. 3b and Fig. S9, compared with the original PGA, PGA–DA exhibits new characteristic peaks at 280 nm (UV–vis) and 6.74 ppm (^1^H NMR), corresponding to the benzene group, which verifies the successful modification of DA. To confirm the presence of adhesive functional components in the hydrogel interface layer, we conducted Fourier-transform infrared spectroscopy with attenuated total reflection (FTIR-ATR) analysis on different hydrogels, including NIPAM-based hydrogel without CNF [NH (w/o CNF)], NIPAM/PGA–DA hydrogel without CNF [NDH (w/o CNF)], NIPAM/zwitterion-based/PGA–DA hydrogel without CNF [NZDH (w/o CNF)] and NZDH. As shown in Fig. 3c and Fig. S10, the characteristic peaks of phenolic hydroxyl groups in the benzene ring and β-glycosidic bonds in CNF appear on the NZDH interface layer, validating the presence of catechol groups and CNF (details discussed in Note S2). Additionally, a new peak emerges at 167.88 eV in NZDHs, which is attributed to the S2p (SO_3_) of PDMAPS, further validating the successful assembly of zwitterionic chains into hydrogel networks (Figs S11 and S12).

To elucidate its temperature-triggered adhesion regulation mechanism, we first directly printed a 2 × 2 NZDH hydrogel precursor interface layer array on the substrate and applied the UV treatment to initiate the crosslinking of hydrogels (Fig. S13, Movie S4). We further compared the optical images and cross-sectional scanning electron microscopy (SEM) images of hydrogel networks under RT and heating conditions. As shown in Fig. 3d, NZDHs are transparent at RT, which is attributed to the extended hydrophilic conformation of PNIPAM chains with a uniform refractive index. Moreover, the adhesive functional groups can form abundant adhesive bonds with the skin (e.g. hydrogen bonds, electrostatic coupling, Michael addition and Schiff base reaction). When heated above the phase-transition temperature, the hydrogen bonds between PNIPAM chains and water molecules undergo rupture, leading to the dehydration of PNIPAM chains and the formation of hydrophobic clusters. The pore size in the hydrogel networks decreases sharply, causing the expulsion of water molecules and denser chain entanglements. Therefore, the adhesive bonds (e.g. hydrogen bonds, electrostatic bonds) formed with the skin are disrupted, resulting in a substantial drop in interface adhesive strength. Meanwhile, the hydrogel interface layer exhibits anisotropic light scattering and appears milky white. Given that the surface temperature of human skin typically ranges from 30 to 34°C, the interface layer requires an appropriate lower critical solution temperature (LCST) to achieve thermally triggered adhesion regulation [44]. Therefore, we adjusted the LCST of hydrogels by incorporating the hydrophilic dimethyl(methacryloyloxyethyl) ammonium propane sulfonate (DMAPS) monomers in hydrogels [45]. As shown in Fig. 3e, compared with that of NDHs (33.1°C), the endothermic peak temperature in heat-flow curves of hydrogels decorated with zwitterionic chains (i.e. NZDHs) shifts to higher values, resulting in an increased phase transition temperature of 35.8°C. To quantitatively evaluate the thermally triggered tunable adhesion of NZDHs, the hydrogel interface layer was heated to different temperatures (e.g. 25°C, 30°C, 35°C, 40°C and 50°C) and adhered to the pigskin substrate for tensile-strength tests. As shown in Fig. 3f and Fig. S14a, the NZDH exhibits excellent adhesion of 27.8 kPa at 25°C. After the temperature is raised to 35°C, the adhesive strength of hydrogels decreases slightly, which is still tightly integrated with the substrate. It is noted that when the temperature is further increased to 40 and 45°C, the adhesion dramatically decreases to 5.30 and 3.94 kPa, respectively, which confirms the superior adhesion-regulation performance of NZDHs.

We further attached the transparent NZDH to skin at RT, realizing a robust-coupling hydrogel/skin interface. When the interface layer is heated to 45°C, the hydrogel turns milky white and can be easily peeled off the skin (Fig. 3g, Movie S5). We also compare the adhesion of NH, NIPAM/zwitterion-based hydrogel (NZH) and NZDH at RT and 45°C (Fig. 3h and Fig. S14b). The results show that due to the modification of DMAPS and DA groups, the initial adhesion of NZDHs is drastically improved. Due to the incorporation of thermoresponsive PNIPAM chains, all three types of hydrogels present tunable adhesion with NZDHs, demonstrating the widest regulation range (>7-fold). To evaluate the stability of adhesion regulation of hydrogels, we conducted cyclic adhesion tests. As shown in Fig. 3i and Fig. S15, during the temperature switching between RT and 45°C, the adhesive strength of NZDH interface layers dramatically changes. It is highlighted that the regulation range reaches ∼10.8 times in the fifth cycle, confirming the adhesion-regulation repeatability of hydrogel interface layers. Moreover, compared with the 3M tape (16.29 kPa), the NZDH also exhibits higher initial adhesive strength (27.8 kPa) (Fig. S16). The mechanical properties of printed NZDH interface layers were also characterized (Fig. S17a, Note S3, Movie S4). The results show that the ultimate tensile strain, fracture stress and elastic modulus of hydrogels are 54%, 7.3 kPa and 17.3 kPa, respectively (Fig. S17b–d). These findings prove the excellent flexibility of NZDH, which facilitates the conformal integration with skin under large-deformation conditions.

Notably, during the monitoring of postoperative flap blood-circulation complications, the hydrogel interface layer needs to be directly attached to flap skin. Therefore, ensuring the biocompatibility of NZDHs is critical. According to ISO 10993-5 (Third edition, 2009-06-01), cytotoxicity tests were performed using human immortalized keratinocytes (HaCaTs) and fibroblasts to evaluate the biocompatibility of hydrogels [46,47]. As shown in Fig. 3j and Fig. S18a, the results show that both HaCaTs and fibroblasts in the hydrogel and control group exhibit normal proliferation, with no obvious signs of apoptosis or necrosis. After 96 h, the relative cell viability and proliferation in the hydrogel group are >99% and >2.2, respectively, comparable to those in the control group (Fig. 3k, Figs S18b, c and S19). The hydrogel samples were further sutured onto the depilated skin of Sprague Dawley rats to evaluate the *in vivo* biocompatibility of NZDHs (Fig. 3l, Fig. S20). No infiltration of inflammatory cells is observed in either group, with the inflammation level being maintained below 1 through blinded pathological histological evaluation (Fig. S20b). We also conducted cytokine level assays to quantify the inflammatory effect of hydrogel layers. Specifically, after applying the hydrogel patch to the depilated dorsal skin surface for 72 h, full-thickness skin tissue samples were harvested from the wound site. The concentrations of IL-1β and IL-6, which were regarded as typical pro-inflammatory cytokines, were then measured in the supernatant of tissue homogenates. The results show that the levels of IL-1β and IL-6 in the hydrogel group are comparable to those in the control group, suggesting no pro-inflammatory effect (Fig. S20c and d). This further confirms the excellent biocompatibility of hydrogel interface layers.

### Working principle and performances of the hydrogel biosensor

**Figure 4.**
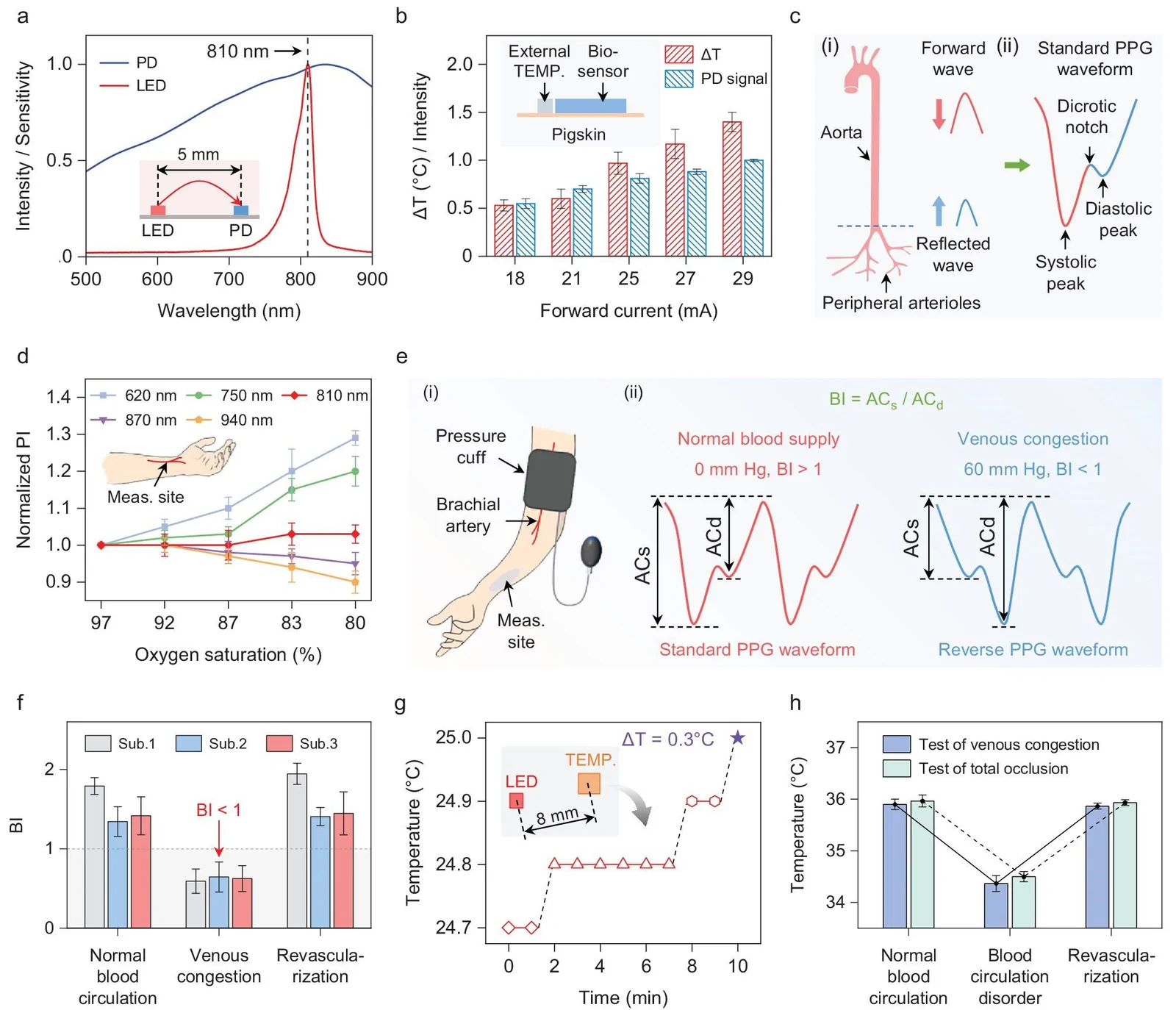
Performance characterizations of the hydrogel biosensor. (a) Relative emission spectra of the LED and spectral sensitivity of the PD. The inset illustrates schematically that the L–P distance is 5 mm. (b) Temperature variations of the pigskin measured by an external temperature sensor, and the normalized intensity of the PD signal, during the 5-min operation of the biosensor under a range of forward currents (mean ± SD; *n* > 30 per group). The inset shows the relative positions of the external temperature sensor and the biosensor on the pigskin schematically. TEMP., temperature sensor; PD signal, the reflective light signal received by PD. (c) The process of pulse wave formation (i) and a standard PPG waveform (ii). (d) Results of PI calculated from different light sources under a range of oxygen saturation (mean ± SD; *n* = 3 per group). The inset shows the sensor being placed on the left wrist to collect PPG signals from the radial artery schematically. Meas. site, measurement site. (e) Measurement setup for inducing venous congestion by a pressure cuff (i), and morphological changes in PPG waveforms before and after venous congestion (ii). (f) BI measurements of three healthy subjects under different blood circulation conditions (mean ± SD; *n* > 30 per group). Sub., subject. (g) Temperature rises of the pigskin measured by the temperature sensor integrated in the front end during the 10-min operation of the biosensor. The inset shows the relative positions of the LED and the temperature sensor schematically. (h) Changes in skin temperature of a healthy subject under different tests (mean ± SD; *n* = 3 per group).

For collecting PPG signals, an LED and a PD are used for light‐emitting and receiving reflective light signals, respectively (Fig. S21). The peak wavelength of the LED lies near the wavelength of peak sensitivity of the PD, enabling the PD to sensitively detect minor variations of the light signal (Fig. 4a). Considering the depth of light path from the LED to the PD, and the size limitation of the on-skin front end, the distance between the LED and the PD (L–P distance) is designed to be 5 mm (Fig. 4a, inset). With the primary consideration of power consumption, the duty cycle was set to 10% (Fig. S22a). To ensure sufficient signal intensity while minimizing the effect of heating on skin, the forward current of the LED was set at 21 mA (Fig. 4b, Fig. S22b and c).

Figure 4c(i) schematically illustrates the formation of pulse waveform in human arterial vessels. The pulse waveform comes into being under the combined influence of the forward wave and the reflected wave. The forward wave is generated by the pressure wave that arises when the heart ejects blood to the periphery, whereas the reflected wave is caused by the reflection from peripheral arterioles [48–50]. Figure 4c(ii) depicts a standard reflective PPG waveform, where three main morphological characteristics, systolic peak, dicrotic notch and diastolic peak, can be clearly identified [8,49]. Particularly, it is believed that the systolic peak and the diastolic peak are formed by the forward wave and the reflected wave, respectively. Successful recording of standard PPG waveforms, and comparison of the pulse rate (PR) measured by the biosensor and a reference commercial pulse oximeter under breathing and breath-holding conditions verified the biosensor’s capability for PPG signal acquisition (Fig. S23). Furthermore, the biosensor exhibited stable measurement performances even under repeated bending conditions (Fig. S24), and the practical utility of the biosensor was demonstrated through measurements taken from various sites of a healthy adult, as presented in Fig. S25 and Movies S6 and S7.

PI, which is highly correlated with arterial perfusion, can be calculated as the ratio between the alternating current and direct current components of PPG signals (Fig. S26), representing the ratio between pulsatile arterial blood and non-pulsatile blood in the peripheral tissue [51]. However, due to the differential absorption of oxyhemoglobin (HbO_2_) and deoxyhemoglobin (Hb) for the same light source, PI varies in response to corresponding changes in blood oxygen content, even when the arterial perfusion remains constant [39]. It is highlighted that the wavelength of 810 nm serves as an isosbestic point for HbO_2_ and Hb, where the absorption coefficients of these two hemoglobins are equal [39–42], thus the PI measured remains unaffected by blood oxygenation variations, only relying on the perfusion state (Fig. 4d). We also constructed a venous congestion model induced by cuff pressure on the healthy adult subject to illustrate the effect of venous congestion on PPG waveforms, as shown in Fig. 4e(i). Under normal blood circulation conditions, PPG signals exhibited a standard waveform, with the amplitude of the systolic peak (AC_s_) being greater than that of the diastolic peak (AC_d_). However, when venous congestion induced by the cuff pressure occurred, PPG signals presented an abnormal waveform (i.e. reverse waveform as we defined), where AC_s_ was lower than AC_d_, as shown in Fig. 4e(ii). Based on this finding, we proposed a new indicator, the balance index (BI), which is defined as


\begin{eqnarray*}
BI = \frac{{AC_{\mathrm{s}}}}{{AC_{\rm d}}},
\end{eqnarray*}

to quantitatively characterize the change of PPG waveforms induced by venous congestion, with BI < 1 indicating the occurrence of venous congestion. This phenomenon may be attributed to the enhanced reflection of peripheral arterioles on blood when venous congestion occurs, leading to a higher reflected wave than the incident wave. A series of venous congestion–revascularization experiments carried out on three healthy adult subjects further demonstrated the efficacy of the BI in detecting venous congestion (Fig. 4f, Fig. S27). The temperature sensor is designed to be placed 8 mm away from the LED to reduce the influence of heating on the actual skin temperature measurement (Fig. 4g). The influence of blood circulation disorders including venous congestion and total occlusion of both veins and arteries on the decrease of skin temperature is demonstrated in Fig. 4h and Fig. S28.

### Tests on animal models

**Figure 5.**
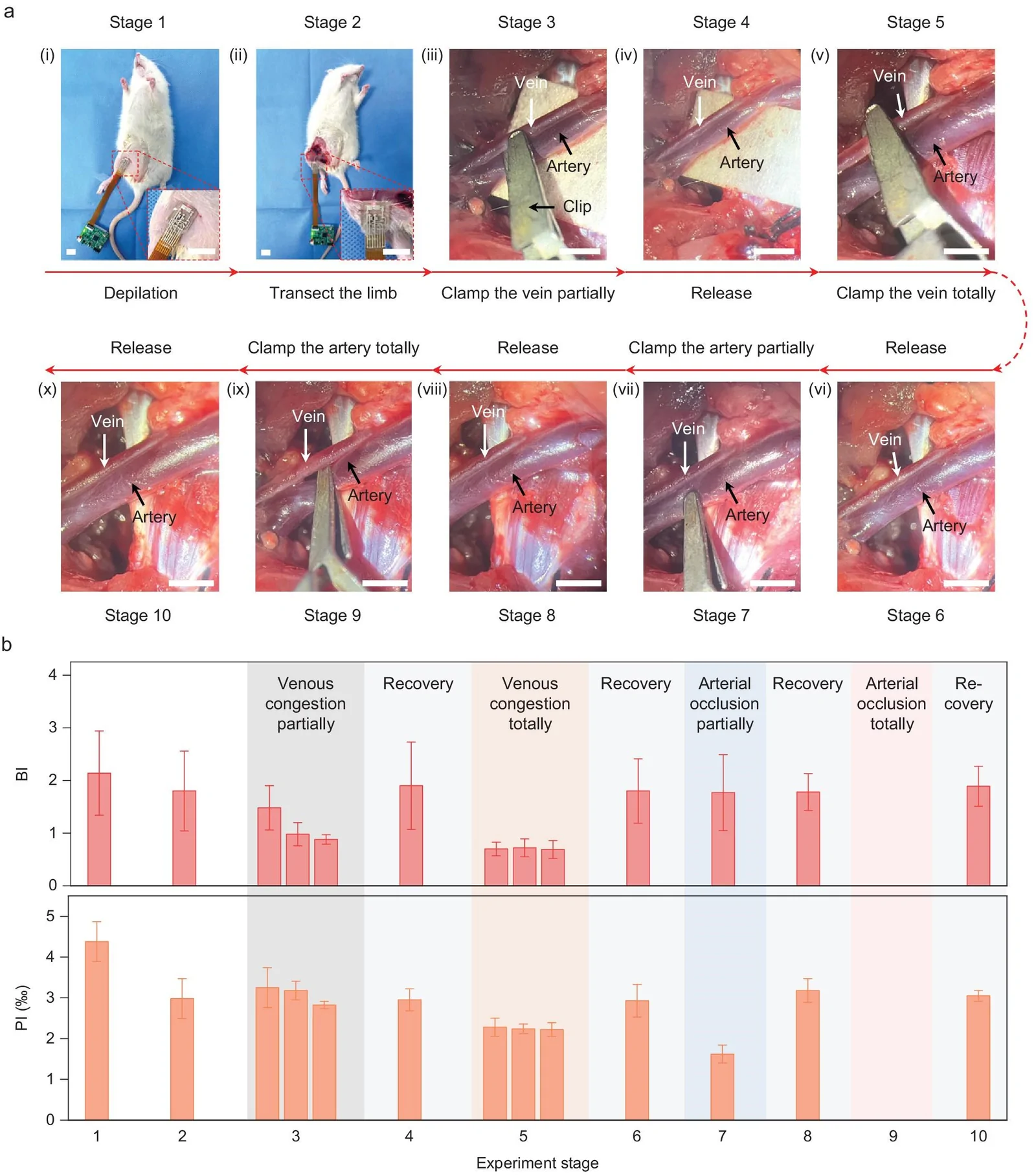
Blood circulation monitoring on an artificial flap model in rats. (a) Experimental protocols of *in vivo* blood circulation monitoring in a rat model. In Stage 1, the biosensor was attached to the shaved hindlimb of the rat (i). In Stage 2, the hindlimb was transected from the rat body (ii), leaving only an artery and a vein. In Stage 3, the hemostatic clip was applied to the vein (iii), inducing partial venous congestion. In Stage 4, the hemostatic clip was released from the vein (iv). In Stage 5, the hemostatic clip was applied to the vein (v), inducing total venous congestion. In Stage 6, the hemostatic clip was released from the vein (vi). In Stage 7, the hemostatic clip was applied to the artery (vii), inducing partial arterial occlusion. In Stage 8, the hemostatic clip was released from the artery (viii). In Stage 9, the hemostatic clip was applied to the artery (ix), inducing total arterial occlusion. In Stage 10, the hemostatic clip was released from the artery (x). (b) Measurements of BI and PI during various stages of the procedure (mean ± SD; *n* > 30 per group). Scale bars, 10 mm (a(i–ii)) and 5 mm (a(iii–x)).

We further investigate the correlation between the BI/PI with different blood circulation states, based on artificial flap models of Sprague Dawley rats. Experimental processes consist of 10 stages, as illustrated in Fig. 5a, and corresponding measurements of BI and PI are shown in Fig. 5b. Various artificial blood circulation complications including partial venous congestion, total venous congestion, partial arterial occlusion and total arterial occlusion were induced by applying a vascular clip on the artery or vein. Owing to stable adhesion of the hydrogel interface, the biosensor can always be attached firmly to the rat skin surface, ensuring the acquisition of high-quality PPG signals at all stages.

The measurements in Stage 1 depict the raw levels of BI and PI before the operation, in which BI is 2.14, and PI is 4.38‰ (Fig. 5b). When the hindlimb of the rat was transected, leaving only the femoral artery and its accompanying vein to simulate the vascular conditions of free flaps, BI and PI dropped to 1.8 and 2.98‰, respectively, and were considered as baseline values for surgical procedures (Stage 2, Fig. 5b). Changes in BI from Stage 3 to Stage 6 illustrate that venous congestion, including partial venous congestion and total venous congestion, leads to the decrease of BI to below 1. Specifically, in the case of partial venous congestion, the BI gradually decreased below 1, with values of 1.48, 0.98 and 0.88 at 1, 3 and 5 min after the onset of congestion, respectively (Stage 3, Fig. 5b). In contrast, for total venous congestion, the BI rapidly decreased below 1, with values of 0.7, 0.72 and 0.69 at 1, 3 and 5 min after the onset of congestion, respectively (Stage 5, Fig. 5b). When the clip was released for about 2 min for blood circulation recovery, BI increased to baseline levels (Stage 4 and Stage 6, Fig. 5b). The variations of BI from Stage 7 to Stage 10 illustrate that partial arterial occlusion does not cause BI to decrease below 1 (Stage 7, Fig. 5b), whereas total arterial occlusion leads to the disappearance of PPG signals such that the BI cannot be calculated (Stage 9, Fig. 5b, Fig. S29). The measurements from Stage 3 to Stage 8 reveal that both venous congestion and partial arterial occlusion give rise to the decrease of PI, with partial arterial occlusion causing a more apparent decrease. It is noted that when venous congestion occurs, BI is less than 1, whereas when partial arterial occlusion occurs, BI is greater than 1. As a result, BI > 1 with PI decreasing is indicative of partial arterial occlusion. When total arterial occlusion occurs, PI cannot be calculated (Stage 9 and Stage 10, Fig. 5b). By analyzing BI, PI and skin temperature, we established the principles for detecting and differentiating different blood circulation complications of free flaps. Specifically, BI < 1 and low values of skin temperature indicate venous congestion. BI > 1 and obvious decrease in both PI and skin temperature indicate arterial spasm (i.e. partial arterial occlusion). The disappearance of PPG waveforms to the extent that BI and PI cannot be calculated indicates arterial occlusion.

### Clinical studies for detecting and differentiating blood circulation complications

**Figure 6.**
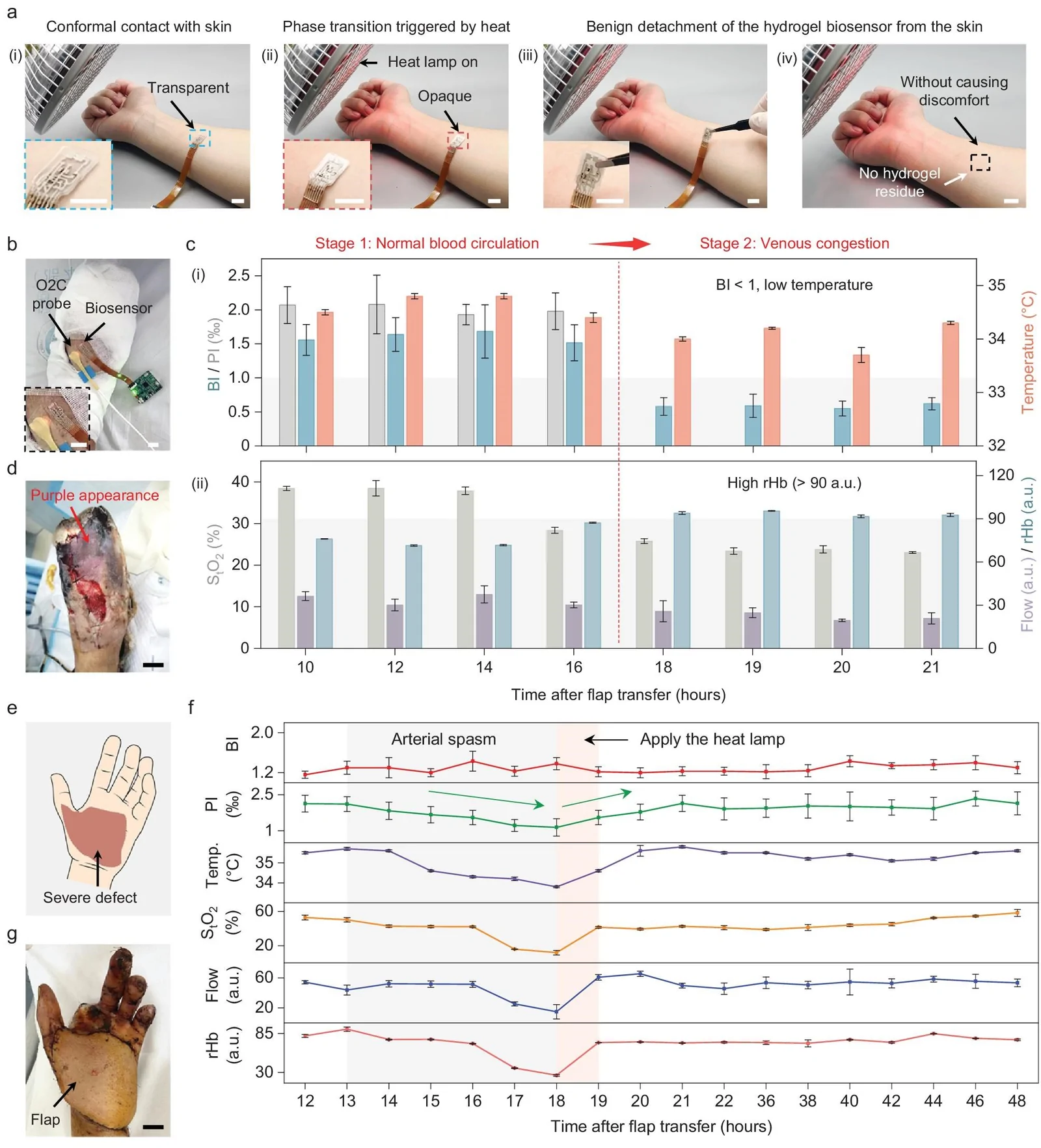
Monitoring venous and arterial complications by the hydrogel biosensor. (a) Demonstration of thermally triggered tunable adhesion properties of the hydrogel biosensor. (b–d) Venous congestion monitoring in Case 1. (b) Photographs of postoperative measurements on the transferred flap by both biosensor and O2C probe (inset shows details). (c) Measurement results by the biosensor and O2C (mean ± SD; *n* > 30 per group). (d) Photograph of the flap with purple skin color. (e–g) Arterial spasm monitoring in Case 6. (e) Schematic illustration of the soft tissue defect in the left palm of the hand. (f) Measurement results by the biosensor and O2C (mean ± SD; *n* > 30 per group). Temp., temperature. (g) Photograph of the flap with normal skin color. Scale bars, 10 mm (a, b, d, g).

Studies on the applicability of the biosensor for detecting and differentiating blood circulation complications in postoperative free flaps include comparisons with measurements from clinical assessment and a commercial microcirculation monitoring system O2C (LW 1111, LEA Medizintechnik). The O2C system monitors tissue oxygen saturation (S_t_O_2_), blood flow velocity (Flow), and relative amount of hemoglobin (rHb) of free flaps (Fig. S30). Previous clinical studies have demonstrated that a sudden decrease and/or low values of S_t_O_2_ and Flow (S_t_O_2_ < 15% and Flow < 20 a.u.) are highly predictive of arterial insufficiency, and a >30% increase or high value of rHb above 90 a.u. indicates venous congestion [20,52]. In postoperative monitoring scenarios, both the biosensor and O2C probe were used to collect blood circulation signals. Notably, they were attached to the flap throughout the monitoring period, with surgeons recording signals at 1-h intervals for clinical assessments. Figure S31 outlines the procedures for data analysis based on values of BI, PI and skin temperature measured from the biosensor. Importantly, when the mean value of BI is less than 1, BI and temperature are adopted to evaluate blood circulation complications. When the mean value of BI is otherwise greater than or equal to 1, BI, PI and temperature are used for evaluation (Fig. S32). For clinical tests, we first validated the thermally triggered tunable adhesion properties of hydrogel biosensors on the healthy subject. As shown in Fig.6a and Movie S8, the hydrogel biosensor achieves seamless integration with the subject’s hand. After irradiation by a widely adopted infrared heat lamp in clinical wards, the hydrogel interface layer undergoes the thermally induced phase transition, enabling mild removal from the skin without causing discomfort. Subsequently, we employed the same adhesion modulation strategy in all clinical cases.

In Case 1, a 55-year-old man sustained an open fracture in his right foot. The right latissimus dorsi (LD) free flap was transferred to the exposed site for tissue coverage (Fig. S33a). Continuous postoperative blood circulation monitoring was carried out (Fig. 6b), and representative measurements are presented in Fig. 6c. In Stage 1, the measurements by the biosensor revealed the BI to be above 1 and relatively smooth changes in PI and temperature, indicating normal blood circulation. Correspondingly, all O2C measurements during this stage were within the normal range, and the surgeon also confirmed satisfactory vascular conditions. However, at 18 h after the operation, BI dropped sharply below 1, with the skin temperature also dropping to a low level (∼34°C), leading to the prediction of venous congestion, which agreed with the indication from O2C due to decreased values of S_t_O2 and Flow, and a high rHb (>90 a.u.). This judgment was confirmed by the surgeon at 19 h after the operation, based on the short capillary refill time (<2 s) and the purple-appearing flap (Fig. 6d). Although urgent salvage procedures, such as flap warming, flap tension relief and adjustment of the patient’s lying posture, were carried out from 19 to 21 h after the operation, the measurements from both the biosensor and O2C, and the dark purple appearance of the free flap, all indicated aggravated venous congestion (Fig. S33b and c). Subsequent surgical re-exploration revealed the presence of thrombosis within the entire length of the vein from the anastomosis to the flap, which led to venous congestion.

In Case 2, an anterolateral thigh (ALT) free flap was transferred to the left hand of a 41-year-old man to treat severe burn scars (Fig. S34a–c). Notably, in Stage 1, the biosensor identified mild venous congestion based on a BI of slightly less than 1, whereas the O2C merely indicated a potential blood circulation complication without predicting the specific type of the complication (Fig. S34d). As time progressed to Stage 2, the BI became much less than 1 and the skin temperature was substantially lower than that in Stage 1; we predicted that a more severe venous congestion than in Stage 1 occurred (Fig. S34d). Meanwhile, the rHb value increased above 90 a.u. in this stage, leading to the prediction of venous congestion. During the parallel monitoring by clinical methods, the surgeon identified venous congestion at 32 h after the operation (Fig. S34e), which was later than judgments made by the biosensor and O2C. At approximately 35 h after the flap transfer, re-exploration surgery was performed to address venous congestion. Subsequent measurements by our biosensor and O2C in Stage 3, together with the surgeon’s assessment, collectively confirmed revascularization after the flap salvage operation (Fig. S34d–f). Blood circulation conditions in another three representative cases experiencing venous congestion (Cases 3 to 5) are presented in Figs S35–S37. The venous congestion in those cases was detected by the biosensor in a timely fashion, and was relieved eventually through effective salvage methods, instead of re-exploration surgery.

In addition to venous congestion, the biosensor was also utilized to detect arterial complications. In Case 6, an ALT free flap was transferred to repair the large-area skin defect near the left palm of a 35-year-old man (Fig. 6e, Fig. S38). At about 12 h after the operation, the surgeon determined that there was no blood circulation complications, and all indicators measured by the biosensor and O2C were within the normal range (Fig. 6f). However, from 13 to 18 h after the operation, PI and skin temperature decreased continuously. Notably, during this stage, the BI remained above 1. Thus, we predicted that the flap suffered acute arterial spasm (i.e. partial arterial occlusion), which was also consistent with O2C prediction of arterial spasm because of the overall downward trend of S_t_O_2_, Flow and rHb (Fig. 6f and g). This symptom was effectively relieved after using a heat lamp to improve the blood circulation for about half an hour, as evidenced by increased PI, skin temperature and measurements from O2C. Subsequent continuous monitoring proved that the flap did not suffer from any blood circulation complications again and the flap finally survived. Figure S39 shows the receiver operating characteristic (ROC) curves for the detection of venous congestion and arterial spasm. The area under each ROC curve (AUC) is greater than 0.8, indicating the effectiveness of the biosensor.

Similarly, an ALT free flap was transferred to cover severe soft tissue defects in the right foot of a 68-year-old woman (Case 7, Fig. S40a). The arterial occlusion was noticed at 10 h after transfer because of a dark appearance and loss of pulse rhythm of PPG signals (Fig. S40b–d). This arterial complication was further confirmed by the prolonged capillary refill time (>2 s) and continuous low levels of S_t_O_2_ (∼10%) and Flow (<15 a.u.), as shown in Fig. S40e. Subsequently, the second operation was performed at about 15 h after transfer, and the flap was saved eventually, as shown in Fig. S40f and g.

These results demonstrated that the biosensor could accurately detect and differentiate different blood circulation complications of free flaps, including venous congestion, arterial spasm and arterial occlusion. In addition, representative cases without any postoperative blood circulation complications were also monitored by the biosensor (Cases 8 to 11, Figs S41–S44, Movie S9), in which the BI was consistently greater than 1, and PI and temperature changed in a stable manner. These cases further demonstrated the practical utility of the biosensor, as there were no false-positive results observed throughout the monitoring process. Both the aforementioned adhesion tests and clinical case data confirm that this hydrogel biosensor maintains superior interface adhesion regulation properties while consistently acquiring high-quality signals.

## CONCLUSION

We have reported a soft biosensor with printable responsive hydrogel interfaces that can achieve accurate detection and differentiation of blood circulation complications in postoperative free flaps. Importantly, we developed a printable hydrogel with tunable adhesion to serve as the universal biosensor interface with human skin. The hydrogel interface layers can maintain superior adhesion and high-fidelity signal acquisition during on-skin measurements while exhibiting low adhesion after monitoring to avoid wound damage. Furthermore, to meet the complex requirements of system integration, it is required to achieve high-efficiency customized patterning of hydrogel interfaces. By modifying CNFs to regulate the rheological properties of hydrogel inks, we achieved direct printing with a linewidth precision below 720 µm and patterned hydrogel interface layers of the biosensor within 30 s. Due to the decoration of thermoresponsive monomers and adhesive functional groups, the NZDH exhibited superior initial adhesion (27.8 kPa) and broad adhesion-regulation range (10.8-fold). Although the focus of this study is on the characterizations of tunable adhesion on flap/human skin, the responsive hydrogel interfaces can also enable tunable adhesion on various wet tissues, such as porcine intestine, stomach, liver and heart. As shown in Figs S45 and S46, the NZDH can tightly couple with porcine tissues, demonstrating excellent adhesion at RT (intestine: 9.5 kPa; stomach: 8.8 kPa; liver: 5.5 kPa; heart: 2.4 kPa) while showing dramatically attenuated adhesion at 45°C (intestine: 2.3 kPa; stomach: 1.1 kPa; liver: 0.9 kPa; heart: 0.6 kPa), facilitating the benign disassembly. Therefore, the NZDH can be extended as a universal adhesion-tunable bioelectronic interface for on-skin and implantable system integration. Meanwhile, the printable NZDH inks enable rapid high-precision patterning of customizable hydrogel interface layers, substantially enhancing the manufacturing efficiency of hydrogel electronic systems. We further performed a comparative analysis between NZDHs and existing hydrogel interfaces (Table S2). It is emphasized that the NZDH demonstrated exceptional performances, including strong initial adhesion, wide-range tunable adhesion and ultra-low modulus, outperforming current hydrogel interfaces, particularly directly printable properties.

Notably, the 810 nm light source proposed is isosbestic for both HbO_2_ and Hb, presenting the unique merit of accurately calculating PI without the influence of blood oxygenation. Besides, we proposed BI as the indicator for detecting venous congestion. Importantly, venous congestion can transform the standard PPG waveform into a reverse waveform, thereby reducing BI below 1. This phenomenon may be attributed to the enhanced reflection of peripheral arterioles. Specifically, when there is no blood circulation disorder in peripheral tissues, blood flows from arterioles through capillary networks into veins, and subsequently returns to the right atrium, completing the circulatory cycle. In this state, vascular resistance within the capillary and venous systems remains low, resulting in minimal blood reflection from the peripheral arterioles. However, when venous obstruction occurs, venous outflow is impeded, leading to blood accumulation within the venous system and the development of venous congestion. This markedly increases vascular resistance in the capillary and venous beds, thereby enhancing the reflection of peripheral arterioles on blood. Under such conditions, the reflected wave becomes higher than the incident wave, leading to the formation of reverse PPG waveforms [48–50]. Tests on healthy subjects and animals proved the feasibility of these indicators in detecting various blood circulation conditions, and the following criteria have been established and validated: (i) BI < 1 and low values of skin temperature indicate venous congestion; (ii) BI > 1 and obvious decrease in both PI and skin temperature indicate arterial spasm (i.e. partial arterial occlusion); and (iii) the loss of PPG waveforms indicates total arterial occlusion.

We have also validated the performances of the biosensor in clinical cases. All representative clinical cases are summarized in Table S3, demonstrating the capability of the biosensor to detect and differentiate venous and arterial complications using BI, PI and skin temperature. When compared with clinical assessment methods, the biosensor possesses the advantages of objective results, accuracy and rapid response. In contrast to the O2C system, the biosensor presents the merits of tunable adhesion to avoid flap damage, low cost (Tables S1 and S4), portability and wireless data transmission. Moreover, the biosensor enables monitoring of multiple physiological indicators using a minimal number of optoelectronic components, while its front end features tunable adhesion properties, demonstrating unique advantages over other existing on-skin PPG sensors (Table S5). Importantly, the biosensor has great potential for integration with other sensing modalities to provide a more comprehensive evaluation of blood circulation states of free flaps. For instance, it can be combined with a laser Doppler sensor to achieve synchronized monitoring of PPG and blood flow signals. Moreover, the biosensor can also integrate multiple LEDs and PDs to acquire PPG and NIRS signals, enabling the calculation and monitoring of oxygenation metrics (e.g. blood oxygen saturation and tissue oxygen saturation) and hemoglobin-related information (e.g. changes in hemoglobin concentration). In addition, given that the 810 nm light source has a limited penetration depth into tissue (∼4.2 mm), the biosensor is more suitable for monitoring thin free flaps [53,54]. Overall, the printed thermoresponsive hydrogel can be adopted as a universal interface in flexible electronics for healthcare applications, and this biosensor represents an innovative and effective method for detecting and differentiating blood circulation complications.

## METHODS

### Synthesis of PGA–DA

Firstly, a 2-(*N*-morpholino)ethanesulfonic acid (MES) buffer solution (0.1 M, pH 5.5) was prepared by dissolving 18 mmol MES and 9 mmol NaCl in 180 mL of deionized (DI) water, followed by pH adjustment using acetic acid. Subsequently, 8 g of PGA was dispersed in the MES buffer under continuous stirring, then 20 mmol EDC, 20 mmol *N*-hydroxysuccinimide (NHS) and 20 mmol ascorbic acid were sequentially added, and the mixture was stirred for 2 h. Next, 20 mmol DA was dissolved in 20 mL of MES buffer, degassed under a nitrogen atmosphere, and added dropwise to the reaction mixture. The solution was stirred under nitrogen for 12 h to obtain the crude product. Purification was performed by dialysis against DI water using a cellulose membrane (MWCO: 1000 Da, Viskase, USA), followed by lyophilization (SJIA-10N, Ningbo Shuangjia) to yield PGA–DA. Finally, the synthesized PGA–DA was stored at −20°C for further use.

### Preparation of hydrogel inks and NZDH interface layers

The hydrogel precursor solution was prepared by ultrasonically dissolving 18.6 wt% NIPAM in DI water. Subsequently, PGA–DA, DMAPS, PEGDMA and α-ketoglutaric acid were sequentially added to the mixture at mass ratios of 0.05, 0.1, 0.0025 and 0.02 times of the NIPAM, respectively. Finally, the mixture was degassed and vigorously stirred to obtain a homogeneous precursor solution. Meanwhile, the CNF suspension was prepared by dispersing CNF powders in 2 mL of deionized water. The hydrogel ink was then formulated by mixing the precursor solutions with CNF suspensions under mechanical stirring for 30 min, followed by centrifugal degassing. To fabricate the printed hydrogel interface layer, the ink was loaded into a self-designed direct-ink-writing (DIW) platform. The predefined interface pattern was first designed for the printing software. Subsequently, optimal printing parameters were determined, including nozzle diameter, applied pressure and platform speed, enabling precise printing of hydrogel inks. After the initiation of UV light for 30 min, the patterned inks were photo-crosslinked to obtain the NZDH interface layer.

### Clinical studies

The research protocol was approved by the ethics committee of Union Hospital, Tongji Medical College, Huazhong University of Science and Technology under number IEC 2021-0014. Eleven representative patients were reported in clinical studies, and participated voluntarily with informed consent. Postoperatively, they were in temperature-stabilized wards, and were well treated and attentively cared for to hold stable blood pressure. Clinical assessment, our biosensor and O2C (LW 1111, LEA Medizintechnik) were used for blood circulation monitoring of free flaps. During the monitoring process, all patients were kept in a supine position without any unnecessary motions. Clinical interventions were implemented based on judgments of surgeons with more than 10 years of clinical experience, independent of biosensor and O2C measurements.

### Statistical analysis

The data were expressed as the mean ± SD of at least three samples unless specified otherwise. For statistical analysis, two groups and multiple groups were compared by using the two-sided Student’s *t*-test and one-way analysis of variance (ANOVA) with Tukey’s *post hoc* test between multiple groups, respectively, with *P* < 0.05 being considered as statistically significant.

## Supplementary Material

nwag058_Supplemental_Files

## Data Availability

The data that support the findings of this study are available from the corresponding author upon reasonable request.
