# Supplementary material for: A soft biosensor with printable responsive hydrogel interfaces for detection and differentiation of blood circulation complications
Source: Natl Sci Rev. 2026 Jan 29;13(5):nwag058. doi: 10.1093/nsr/nwag058 (PMC12980341; doi:10.1093/nsr/nwag058)
Supplement: nwag058_Supplemental_Files [file nwag058_supplemental_files.zip › 20260109-Supplementary Materials-Final.pdf]

# Supplementary Materials for

## A soft biosensor with printable responsive hydrogel interfaces for detection and differentiation of blood circulation complications

Yuqi Qiu[1,†], Ganguang Yang[1,†], Zhixin Wang[1], Bo Pang[1], Sen Zhou[1], Qingyang Zheng[1], Tianzhao Bu[1], Jia Tian[2], Bing Xue[3], Junhak Lee[4], Yeonsik Choi[4], Zhouping Yin[1], Changsheng Wu[3,5,6,7*], Yutian Liu[2*], Hao Wu[1,8*]

[1]Flexible Electronics Research Center, State Key Laboratory of Intelligent Manufacturing Equipment and Technology, School of Mechanical Science and Engineering, Huazhong University of Science and Technology, Wuhan 430074, China.
[2]Department of Hand Surgery, Union Hospital, Tongji Medical College, Huazhong University of Science and Technology, Wuhan, Hubei, 430022, China.
[3]Department of Materials Science and Engineering, National University of Singapore, Singapore, 117575, Singapore.
[4]Department of Materials Science and Engineering, Yonsei University, Seoul, 03722, Republic of Korea.
[5]Institute for Health Innovation and Technology, National University of Singapore, Singapore, 117599, Singapore.
[6]Department of Electrical and Computer Engineering, National University of Singapore, Singapore, 119276, Singapore.
[7]The N.1 Institute for Health, National University of Singapore, Singapore, 117456, Singapore.
[8]School of Integrated Circuits, Huazhong University of Science and Technology, Wuhan, Hubei, 430074, China.
[*]Corresponding authors. Email: hwu16@hust.edu.cn; 2016xh0053@hust.edu.cn; cwu@nus.edu.sg.
[†]Equally contributed to this work.

**The PDF file includes:**

Supplementary text

Note S1 to S3

Figure S1 to S46

Table S1 to S5

SI References 1 to 27

**Other Supplementary Materials for this manuscript include the following:**

Movie S1 (.mp4 format) Printing demonstration of hydrogel inks with different weight ratios of CNF/NIPAM.

Movie S2 (.mp4 format) Patterned printing of hydrogel interface layers on the flexible sensing systems.

Movie S3 (.mp4 format) Demonstration of printing two-dimensional and three-dimensional patterns using hydrogel inks.

Movie S4 (.mp4 format) Fabrication of printed hydrogel arrays and hydrogel stretching molds.

Movie S5 (.mp4 format) Thermoresponsive adhesion regulation of printed hydrogel interface layers.

Movie S6 (.mp4 format) Operation of the hydrogel biosensor.

Movie S7 (.mp4 format) Monitoring a healthy subject using the hydrogel biosensor.

Movie S8 (.mp4 format) Benign detachment of the printed hydrogel biosensor triggered by the infrared heat lamp irradiation.

Movie S9 (.mp4 format) Monitoring the anterolateral thigh free flap in case 9.

## Table of Contents

**Supplementary Text**

**Supplementary Notes**


**Note S1.** Material characterizations.
**Note S2.** Detailed discussions on FTIR and XPS spectra of the NZDHs.
**Note S3.** Mechanical Testing.


**Supplementary Figures**


**Figure S1.** Fabrication processes of the on-skin front-end.
**Figure S2.** Photograph of the hydrogel biosensor.
**Figure S3.** Synthesis of the hydrogel inks.
**Figure S4.** Rheological characterization of different hydrogel inks.
**Figure S5.** Comparisons of printing trajectories of hydrogel inks at different platform speeds.
**Figure S6.** Rheological behavior of hydrogel inks with different weight ratios of CNF/NIPAM.
**Figure S7.** Microscope images of printed hydrogel lines under different pressures and platform speeds.
**Figure S8.** Flexible devices fabricated using hydrogel inks.
**Figure S9.** $^1$H NMR spectra of PGA-DA and PGA.
**Figure S10.** FTIR-ATR spectra of NH (w/o CNF), NDH (w/o CNF), NZDH (w/o CNF), and NZDH.
**Figure S11.** XPS analysis of hydrogel interface layers.
**Figure S12.** XPS S2p spectra of NDH (w/o CNF), NZDH (w/o CNF), and NZDH.
**Figure S13.** Direct-printing fabrication of hydrogel interface layers.
**Figure S14.** Comparisons of adhesion regulation performance of thermoresponsive interface layers.
**Figure S15.** Tensile strength of hydrogel interface layers during RT-45°C cycles.
**Figure S16.** Adhesion comparisons of NZDHs and 3M tapes when laminated on the porcine skin.
**Figure S17.** Mechanical characterizations of the NZDH interface layer.
**Figure S18.** Biocompatibility assessment of fibroblasts.
**Figure S19.** Relative cell proliferation of HaCaTs calculated via CCK-8 assays.
**Figure S20.** Inflammatory evaluation of NZDHs.
**Figure S21.** Circuit design of the hydrogel biosensor.
**Figure S22.** Thermal characteristics of the hydrogel biosensor.
**Figure S23.** Signal acquisition capability of the hydrogel biosensor.
**Figure S24.** Measurement reliability of the hydrogel biosensor.
**Figure S25.** Measurements by the hydrogel biosensor on different sites of a healthy subject.

**Figure S26.** The calculation of PI.
**Figure S27.** Effects of venous congestion on PPG waveforms.
**Figure S28.** Effects of venous congestion and total occlusion on skin temperature.
**Figure S29.** Additional data from animal tests.
**Figure S30.** Photographs of the O2C system.
**Figure S31.** Flowchart of the data analysis procedure of the hydrogel biosensor.
**Figure S32.** The customized GUI for signal display and analysis.
**Figure S33.** Photographs from case 1.
**Figure S34.** Monitoring the anterolateral thigh free flap in case 2.
**Figure S35.** Monitoring the anterolateral thigh free flap in case 3.
**Figure S36.** Monitoring the free flap from the wrist in case 4.
**Figure S37.** Monitoring the anterolateral thigh free flap in case 5.
**Figure S38.** Photographs from case 6.
**Figure S39.** ROC curves for the detection of venous congestion and arterial spasm.
**Figure S40.** Monitoring the anterolateral thigh free flap in case 7.
**Figure S41.** Monitoring the medial sural artery perforator free flap in case 8.
**Figure S42.** Monitoring the medial sural artery perforator free flap in case 9.
**Figure S43.** Monitoring the anterolateral thigh free flap in case 10.
**Figure S44.** Monitoring the anterolateral thigh free flap in case 11.
**Figure S45.** Adhesive properties of hydrogel interface layers on different pig tissues.
**Figure S46.** Comparisons of thermoresponsive NZDHs when adhered to different porcine issues.

**Supplementary Tables**

**Table S1.** Comparisons of techniques for blood circulation monitoring of postoperative free flaps.
**Table S2.** Comparisons of hydrogels as interface layers for bioelectronics.
**Table S3.** Summary of representative clinical cases.
**Table S4.** Estimated costs of the hydrogel biosensor developed in this study.
**Table S5.** Comparison of the biosensor with previously reported on-skin PPG sensors.

## Supplementary Text

## Materials

Poly(ethylene glycol methacrylate) (PEGDMA), 3-dimethyl(methacryloyloxyethyl) ammonium propane sulfonate (DMAPS), α-ketoglutaric acid, ascorbic acid, 4-Morpholineethanesulfonic acid (MES), sodium chloride (NaCl), N-Hydroxysuccinimide (NHS), and 1-(3-Dimethylaminopropyl)-3-ethylcarbodiimide hydr-ochloride (EDC) were purchased from Aladdin. Acetic acid was purchased from Sinopharm Chemical Reagent Co., Ltd. N-isopropylacrylamide (NIPAM) was obtained from TCI Shanghai Chemical Industry Development Co., LTD. Polyglutamic acid (PGA) was purchased from D-chem. Cellulose nanofiber (CNF) was purchased from Tianlu Nanotechnology Co., Ltd. All porcine tissues for adhesion experiments were purchased from Tian Chen Frozen Food.

## Rheological characterizations and printing performances of hydrogel inks

The rheological properties of hydrogel inks were characterized using a DHR rheometer (TA Instruments, New Jersey, USA) equipped with a 60.0 mm parallel plate geometry and a 1.0 mm gap. The viscosity-shear rate and stress-shear rate measurements of hydrogel inks, containing varying weight ratios of CNF/NIPAM, were conducted at 25°C with shear rates ranging from 1 to 1000 $s^{-1}$. Printing precision was evaluated by systematically adjusting the extrusion pressure and platform speed during direct ink writing. The printed hydrogel patterns were recorded using optical microscopy, with

linewidth measurements taken at three distinct locations per sample, with reported values representing the mean ± SD.

### Adhesion measurements of NZDH interface layers

The interface bonding strength of hydrogel specimens measuring 25 mm × 25 mm × 1 mm (length × width × thickness) was evaluated through standard uniaxial tensile tests (ASTM F2258). For adhesion regulation experiments, the top surface of hydrogels was affixed to a heating plate using cyanoacrylate adhesive, while the bottom surface of tissues was secured to a T-shaped aluminum fixture. The heating plate was then connected to another T-shaped aluminum fixture. At temperature setpoints, the adhesion strength between the hydrogel and substrate was measured. Before tensile testing, a 5 N preload was applied to the hydrogel/tissue interface for 10 s. All experiments were performed at a constant displacement rate of 50 mm/min. Adhesion strength was calculated as the ratio of maximum load to contact area, with reported values representing the average of at least three independent measurements.

### Biocompatibility evaluation

The biocompatibility of NZDH was assessed through *in vitro* and *in vivo* experiments. For *in vitro* assessment, NZDH samples were immersed in high-glucose DMEM (HG-DMEM; Gibco, USA) for 24, 48, 72, and 96 h, with a blank control group maintained in HG-DMEM alone. The conditioned media were then used to culture HaCaTs and

fibroblasts at 37°C. Cellular viability was qualitatively analyzed via Calcein-AM/ propidium iodide dual fluorescence staining (Yesen Biotechnology, China), while relative cell viability and proliferation were quantitatively evaluated using the CCK-8 assay (Beyotime, China). For *in vivo* evaluation, NZDH implants were subcutaneously embedded in depilated Sprague-Dawley rats. Tissue specimens harvested after 72 hours were paraffin-embedded and sectioned for H&E staining (Bioyear, China). A blinded histopathological evaluation was conducted by the certified pathologist, employing standardized grading criteria encompassing inflammatory cell infiltration density and histomorphological alterations to systematically classify inflammatory responses.

**Animal test procedures**

The animal study protocol was approved by the Animal Care Committee of Huazhong University of Science and Technology under number IEC 2021-S2789. Adult male Sprague-Dawley rats (10 weeks old) were used in our studies. All rats were housed under standard conditions, maintained on a 12-hour light/dark cycle at 22–25 °C, and could get food and water freely. Before the surgery, the rat was anaesthetized via an intraperitoneal injection of 3% pentobarbital sodium solution (30mg per kg body weight). Animal procedures consisted of ten stages.

**Biosensor design and integration**

The front-end was fabricated through the flexible hybrid process, and both the FFC and

the rigid back-end were fabricated by a PCB manufacturer (Shenzhen HONGHUIFU Electronics Technology Co., Ltd.). A rechargeable Li-Po battery was employed to supply 3.7V to the biosensor. To manage the power, a single inductor buck-boost converter (TPS63001, Texas Instruments) was utilized. A MCU (STM32F103C8T6, STMicroelectronics) was selected for system control, and its configuration was accomplished using the STM32CubeMX platform. A LED (IRC81L, BAITE OPTO-ELECTRONIC Co., Ltd.) and a PD (VEMD1060X01, Vishay Semiconductors) were used for generating photocurrent signals. To drive the LED, a NPN plastic-encapsulated transistor (MMBT3904, HXY ELECTRONIC Co., Ltd.) was adopted. A TIA was used to convert the photocurrent signals into time-varying voltage signals, namely PPG signals. The 12-bit ADC of the MCU was used to sample PPG signals. A high-precision digital temperature sensor (TMP117, Texas Instruments) was utilized for collecting temperature signals, with the IIC protocol being applied to transmit temperature signals to the MCU. BLE modules (DX-BT24-T and DX-CP11, DX-SMART Technology Co., Ltd.) were used for real-time wireless data communication. Adhesive tape was used to fix top interconnects of the front-end to exposed copper pads of the FFC, thereby enabling electrical connection between the front-end and the FFC.

**Fabrication of the on-skin flexible hybrid front-end of the biosensor**

The 200-μm-thick cured PDMS (10:1) membrane served as the substrate layer of the front-end. Polyvinyl chloride (PVC) wallpaper was applied to both sides of the PDMS

as the mask. Patterns and through-holes were cut using a carbon dioxide ($CO_2$) laser cutter system (Guangzhou HZZ E-Photo Technology Co. Ltd., ILS-3V), and ECCs were filled into the pre-cut patterns and through-holes. The sample was then heated in an oven at 160°C for 1 hour to establish conductive interconnections. After peeling off the mask from the substrate layer, rigid electronic components were mounted onto the pads of the top interconnection. PDMS (10:1) was poured onto the bottom interconnection and cured in an oven at 70°C for 0.5 hour to form the encapsulation layer. Similarly, PDMS (10:1) was poured onto the top interconnection and cured at 70°C for 0.5 hour to form the separation layer. The sample was then die-cut into the target shape using a custom-made knife mold. Finally, hydrogel was printed onto the separation layer and cross-linked under a UV light source.

**Characterizations of the biosensor performance**

A high-sensitivity spectrometer (HS2000PRO, JINGYIOPTOELECTRONIC Technology Co., Ltd.) was used for emission spectra analysis of the LED. A monochromator (Omni-750i, ZOLIX INSTRUMENTS Co., Ltd.) was used for spectral response analysis of the PD. For each forward current, the biosensor was operated on the pigskin for 5 minutes, with PD signals and temperature signals being recorded by the biosensor and the external temperature sensor, respectively. To investigate effects of changes in oxygen saturation on PI, four different flexible optoelectronic front-ends were fabricated for comparisons with the biosensor. All front-ends were prepared by

the PCB manufacturer (Shenzhen HONGHUIFU Electronics Technology Co., Ltd.) based on the flexible printed circuit board (FPCB) process. In particular, each front-end was configured with a single LED as the light source. Four types of LEDs (IRC62L, IRC75L, IRC87L, and IRC94L, BAITE OPTO-ELECTRONIC Co., Ltd.), which can emit light of different wavelengths, including 620 nm, 750 nm, 870 nm, and 940 nm, were integrated into each front-end, respectively. A medical upper arm blood pressure monitor (A type mercury blood pressure monitor, Jiangsu Yuyue Medical Equipment&Supply Co., Ltd) was used to apply pressure on the brachial artery. Specifically, cuff pressures of 60 mmHg and 180 mmHg were used to induce venous congestion and total occlusion, respectively. To verify the measurement accuracy of PPG signals obtained from the biosensor, a medical-grade transmission-mode finger probe pulse oximeter (Prince-100H, Heal Force Bio-meditech Holdings Limited.) was used to record pulse rates.

**Recording and processing of temperature and PPG signals**

Raw temperature and PPG signals were recorded at a sampling rate of 100 Hz, and were transmitted wirelessly to the laptop PC through BLE modules. Further signal processing was achieved by the GUI that was designed using MATLAB R2021b. To calculate BI, a band-pass finite impulse response (FIR) digital filter with a passband frequency of 0.5-6 Hz were designed to filter raw PPG signals. Subsequently, peak-finding operations were performed on filtered PPG signals to calculate ACs and ACd values.

To calculate PI, a low-pass FIR digital filter with a cutoff frequency of 6 Hz was designed to filter raw PPG signals, and upper and lower peak envelopes were applied to obtain pulsatile and non-pulsatile portions of filtered PPG signals. To obtain the power spectrum of the PPG signals, spectrum analysis based on the Welch method and the Hanning window was applied to the low-filtered PPG signals.

## Supplementary Notes

### Note S1. Material characterizations.

Scanning electron microscopy (SEM) imaging was performed using an SU3900 instrument (HITACHI). Structural characterization of the synthesized PGA-DA was conducted via $^{1}H$ nuclear magnetic resonance spectroscopy (600 MHz AscendTM, Bruker) in deuterated water ($D_2O$), with spectral data processed using MestReNova software. UV-Vis spectrophotometric analysis (Lambda 35, PerkinElmer) confirmed the presence of catechol groups. Chemical composition analysis of hydrogel layers was carried out using Fourier transform infrared spectroscopy (Nicolet iS50, Thermo Fisher) with a germanium-attenuated total reflectance (ATR) crystal at 55°C and X-ray photoelectron spectroscopy (AXIS-ULTRA DLD-600W, Kratos).

### Note S2. Detailed discussions on FTIR and XPS spectra of the NZDHs.

We characterized the FTIR-ATR spectra of NH (w/o CNF), NDH (w/o CNF), NZDH (w/o CNF), and NZDH. Compared with NH (w/o CNF), the new peak at 1338 $cm^{-1}$ in NDH (w/o CNF) and NZDH (w/o CNF) was attributed to the bending vibration of phenolic hydroxyl groups (-OH) in the benzene ring. After the addition of CNF, the peak of -OH shifted to 1321 $cm^{-1}$ in NZDHs due to hydrogen bonding between NZDH networks and CNF. Besides, a new peak at 896 $cm^{-1}$ in NZDHs was assigned to the β-glycosidic bonds in CNF. Furthermore, the NZDH (w/o CNF) exhibited a new peak at 1040 $cm^{-1}$, corresponding to the S-O stretching vibration of $-SO_3$ groups, which was

absent in NH (w/o CNF) and NDH (w/o CNF). Notably, the introduction of CNF in NZDHs led to an overlapping peak at 1055 $cm^{-1}$, attributed to the C-O stretching vibration of primary alcohols in CNF, which interfered with the characteristic $-SO_3$ peak of zwitterionic chains. To confirm the presence of a zwitterionic network in NZDHs, XPS spectra of NDH (w/o CNF), NZDH (w/o CNF), and NZDH were analyzed. The appearance of new peaks at 168.88 eV and 167.88 eV in NZDH (w/o CNF) and NZDH, respectively, assigned to S2p ($SO_3$) of DMAPS, further verified the successful incorporation of zwitterionic chains into the hydrogel network.

**Note S3. Mechanical Testing.**

All hydrogel samples were printed in a dog-bone geometry with standardized dimensions of 25 mm (gauge length) × 9 mm (width) × 1 mm (thickness). Uniaxial tensile testing was performed using the testing system (ZQ-990LA-2, Fengbao instrument) at a constant loading speed of 100 mm/min. The elastic modulus was determined from the linear elastic region of stress-strain curves, while the ultimate tensile strength was calculated as the maximum stress before fracture.

## Supplementary Figures

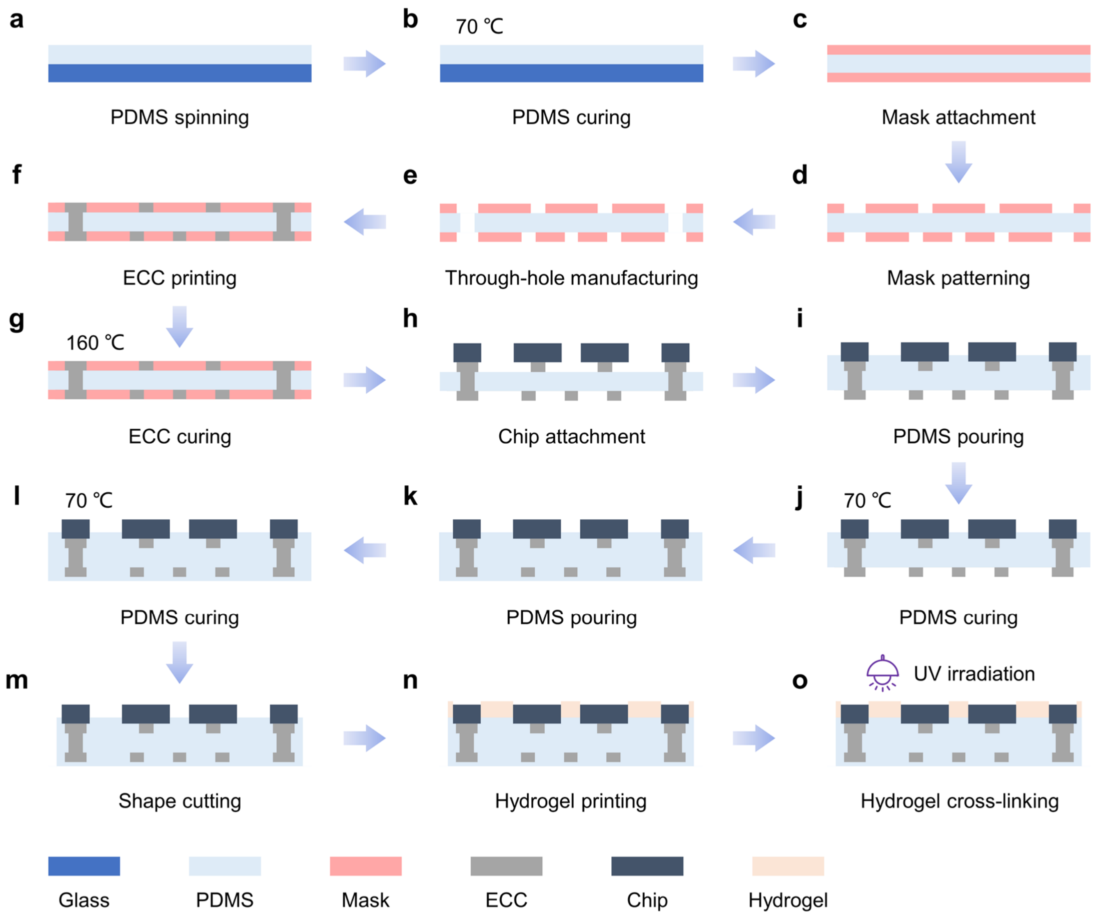


**Figure S1.** Fabrication processes of the on-skin front-end. (a) Spin coating of PDMS on the glass substrate. (b) Curing PDMS at 70 °C and using it as the substrate layer. (c) Attaching masks on the PDMS. (d) Cutting patterns for masks using laser. (e) Punching through holes in masks and PDMS using laser. (f) Coating uncured ECC for PDMS. (g) Curing ECC at 160 °C. (h) Peeling off masks and attaching electronic components to PDMS. (i-j) Pouring PDMS and curing it at 70 °C to fabricating the separation layer. (k-l) Pouring PDMS and curing it at 70 °C to fabricating the encapsulation layer. (m) Cutting the target shape using a knife mold. (n) Printing hydrogel on the separation layer. (o) Cross-linking of the hydrogel.

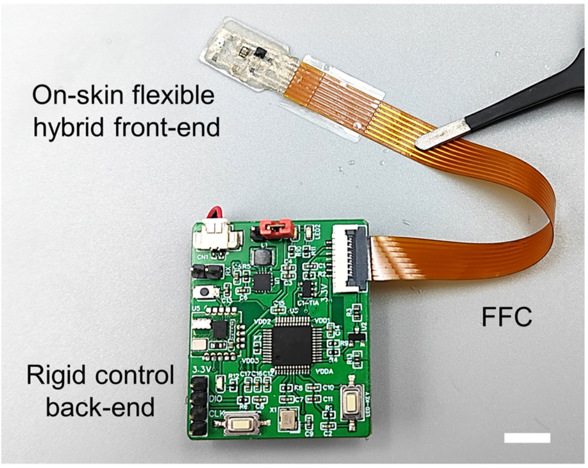


**Figure S2.** Photograph of the hydrogel biosensor. Scale bar, 10 mm.

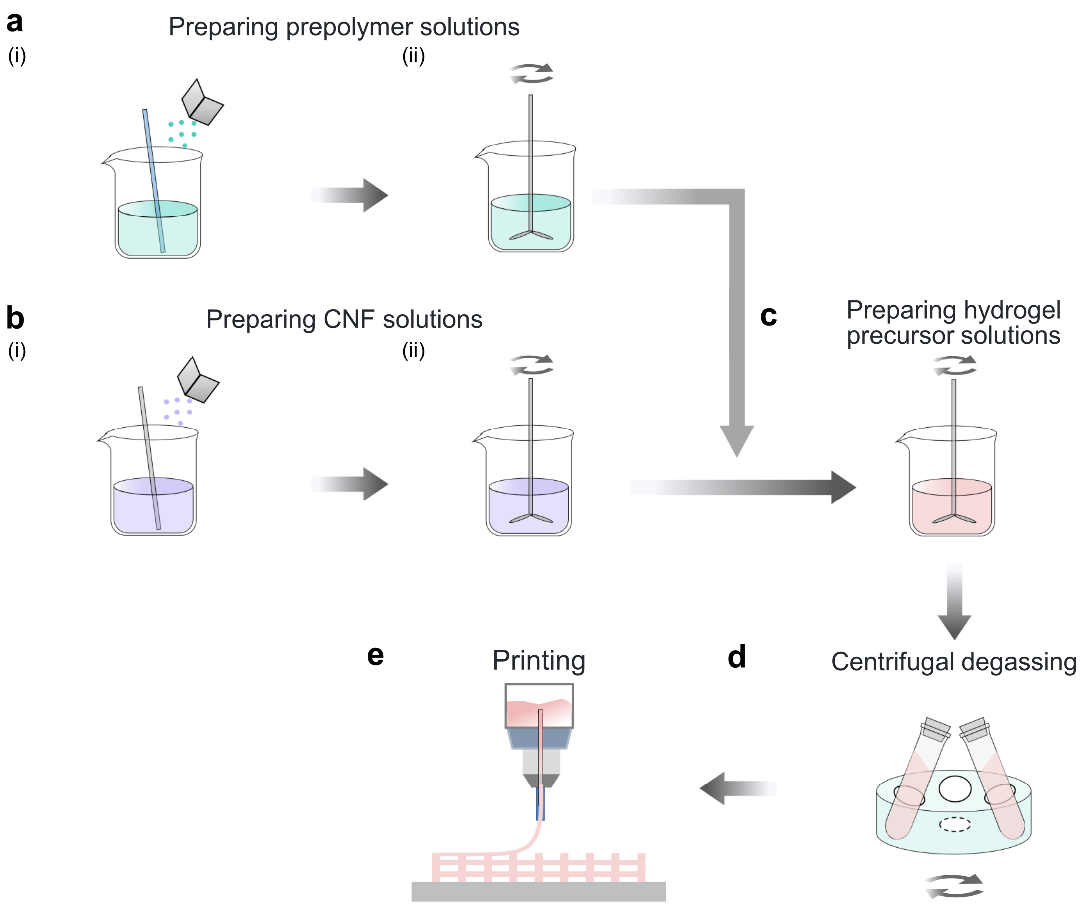


**Figure S3.** Synthesis of the hydrogel inks. (a-b) Preparation of hydrogel prepolymer solutions (a) and CNF solutions (b). (c-d) Mixing and centrifugal degassing of hydrogel precursor inks. (e) Direct printing of hydrogel inks.

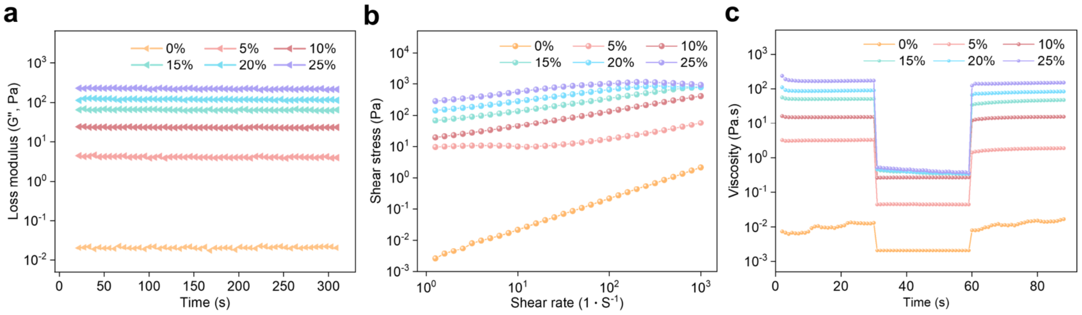


**Figure S4.** Rheological characterizations of different hydrogel inks. (a-c) Loss modulus (G")-time (a), shear stress-shear rate (b), three-interval thixotropy test (3ITT) curves (c) of hydrogel inks with CNF/NIPAM at weight ratios of 0%, 5%, 10%, 15%, 20%, and 25%, respectively.

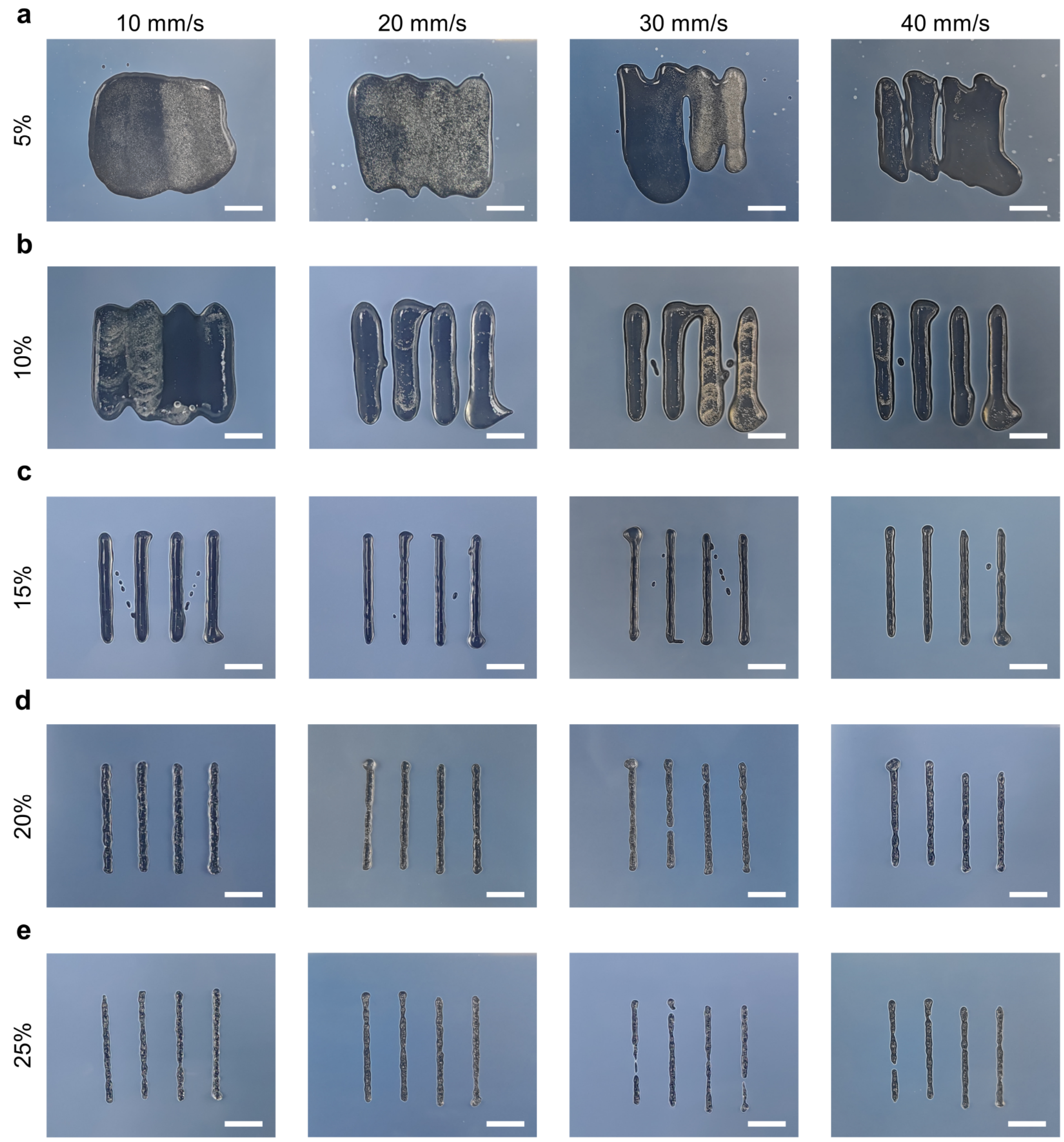


**Figure S5.** Comparisons of printing trajectories of hydrogel inks at different platform speeds. (a-e) Photographs of corresponding printed hydrogel lines with CNF/NIPAM at weight ratios of 5% (a), 10% (b), 15% (c), 20% (d), and 25% (e), respectively. Scale bars, 10 mm (a-e).

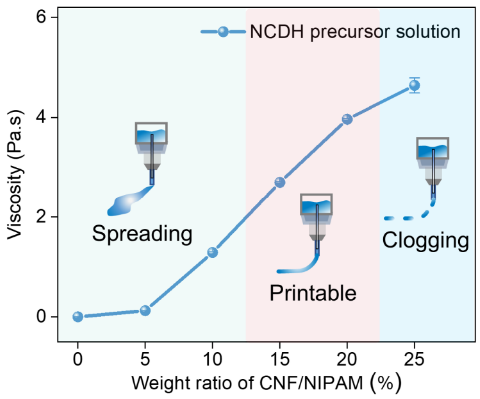


**Figure S6.** Rheological behavior of hydrogel inks with different weight ratios of CNF/NIPAM. At low weight ratios (<10%), the ink demonstrates excessive fluidity, leading to diffusion upon deposition. Optimal printability is achieved within the 15-20% range, while higher weight ratios (>25%) result in nozzle clogging due to increased viscosity.

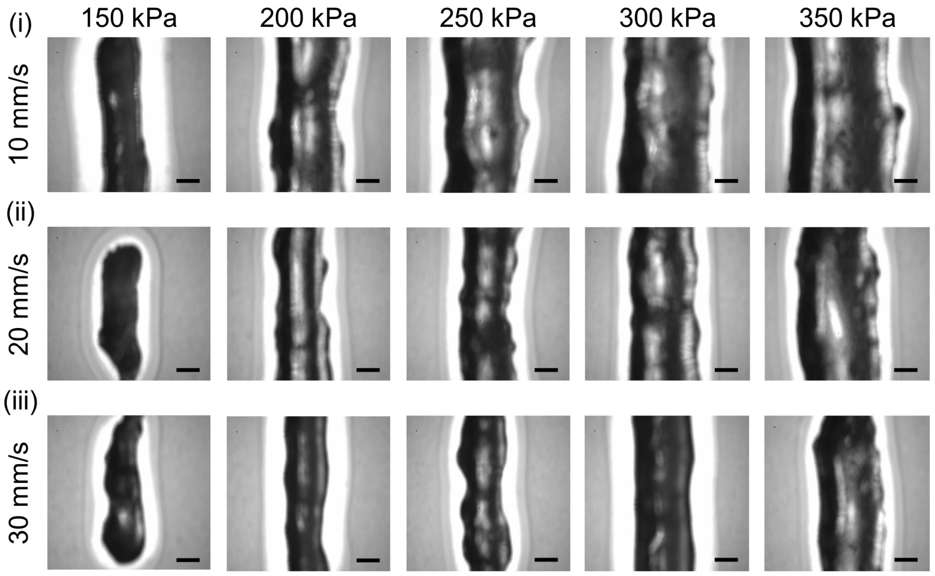


**Figure S7.** Microscope images of printed hydrogel lines under different pressures and platform speeds. Scale bar, 500 μm.

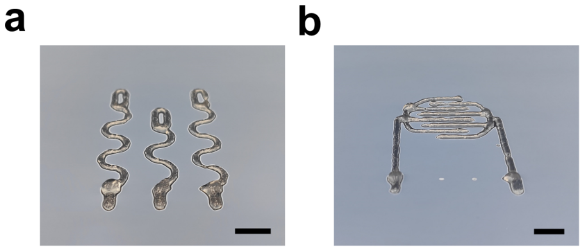


**Figure S8.** Flexible devices fabricated using hydrogel inks. (a-b) Photographs of printed hydrogel electrodes (a) and capacitive sensors (b). Scale bars, 10 mm (a, b).

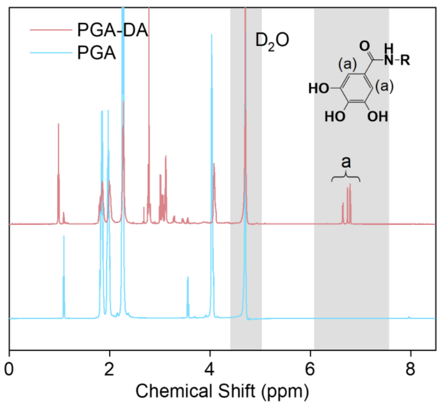


**Figure S9.** $^1H$ NMR spectra of PGA-DA and PGA.

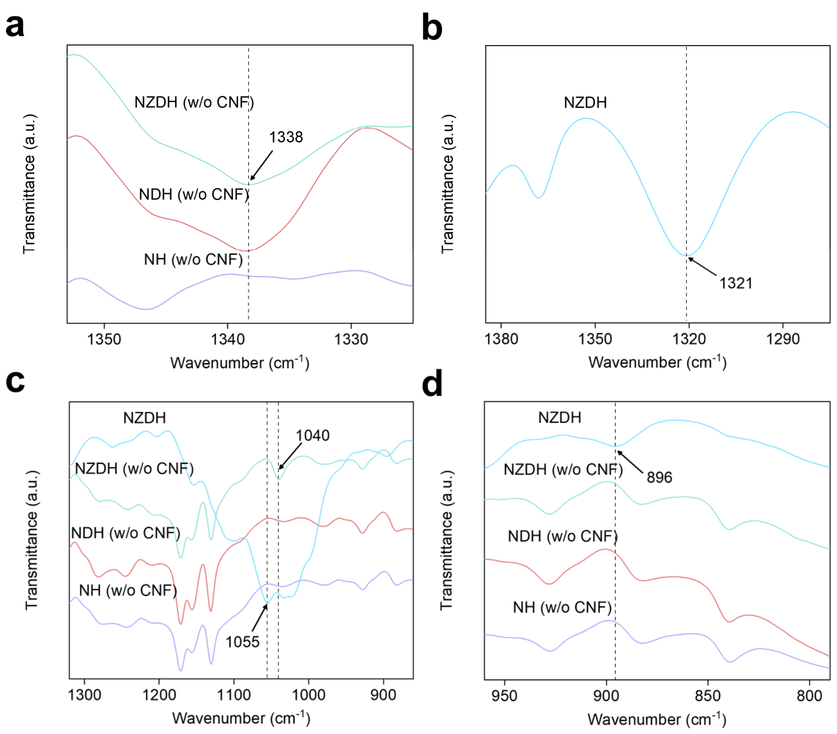


**Figure S10.** FTIR-ATR spectra of NH (w/o CNF), NDH (w/o CNF), NZDH (w/o CNF), and NZDH. (a-d) Magnified peaks at 1338 $cm^{-1}$ (a), 1040 $cm^{-1}$ (c), and 896 $cm^{-1}$ (d), which are assigned to the bending vibration of phenolic hydroxyl groups (-OH) in the benzene ring, S-O stretching vibration in $-SO_3$ groups, and β-glycosidic bonds in CNF, respectively. The peak of phenolic hydroxyl groups (-OH) in NDZHs shifts to 1321 $cm^{-1}$ (b) due to the formation of hydrogen bonds between CNF and hydrogel networks. The peak at 1055 $cm^{-1}$ (c) belongs to the C-O stretching vibration of primary alcohols in CNF.

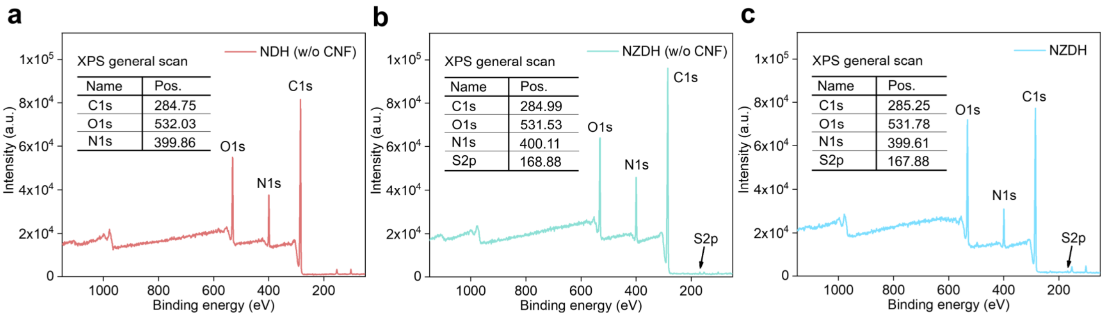


**Figure S11.** XPS analysis of hydrogel interface layers. (a-c) XPS general scan spectra of NDH (w/o CNF) (a), NZDH (w/o CNF) (b), and NZDH (c).

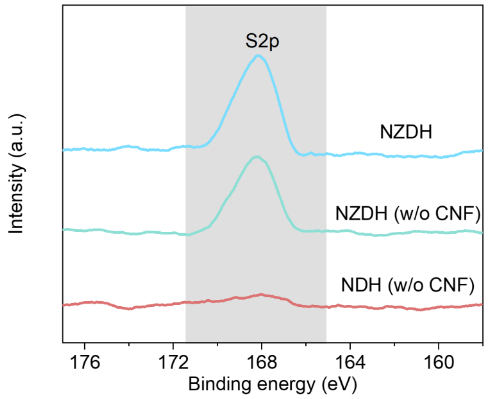


**Figure S12.** XPS S2p spectra of NDH (w/o CNF), NZDH (w/o CNF), and NZDH.

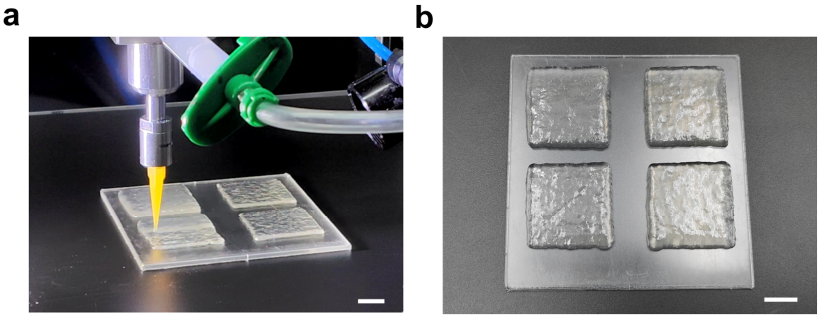


**Figure S13.** Direct-printing fabrication of hydrogel interface layers. (a-b) Photographs of hydrogel interface layers for adhesion tests during (a) and after (b) printing. Scale bars, 10 mm (a, b).

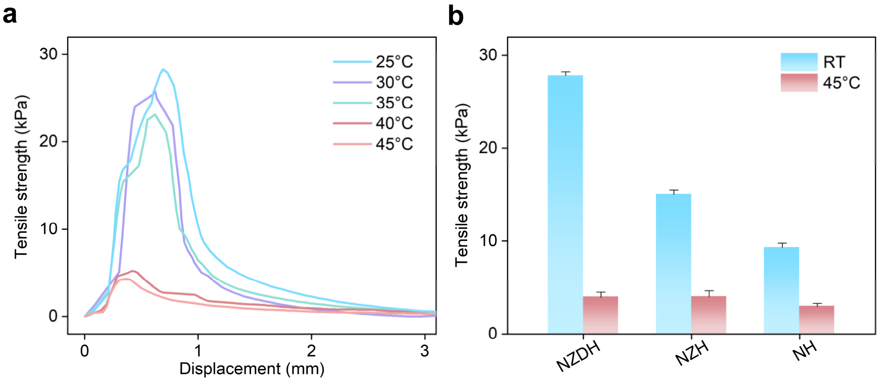


**Figure S14.** Comparisons of adhesion regulation performance of thermoresponsive interface layers. (a) Strength-displacement curves of NZDHs when affixed to the pigskin at different temperatures. (b) Adhesion strength of thermoresponsive hydrogels with different compositions at 45°C and RT, respectively.

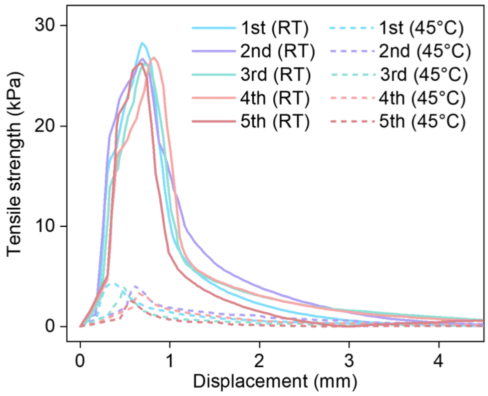


**Figure S15.** Tensile strength of hydrogel interface layers during RT-45°C cycles.

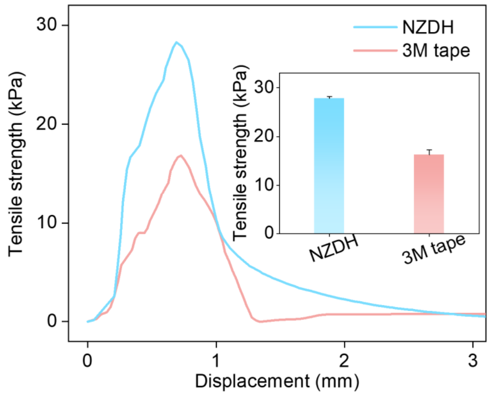


**Figure S16.** Adhesion comparisons of NZDHs and 3M tapes when laminated on the porcine skin.

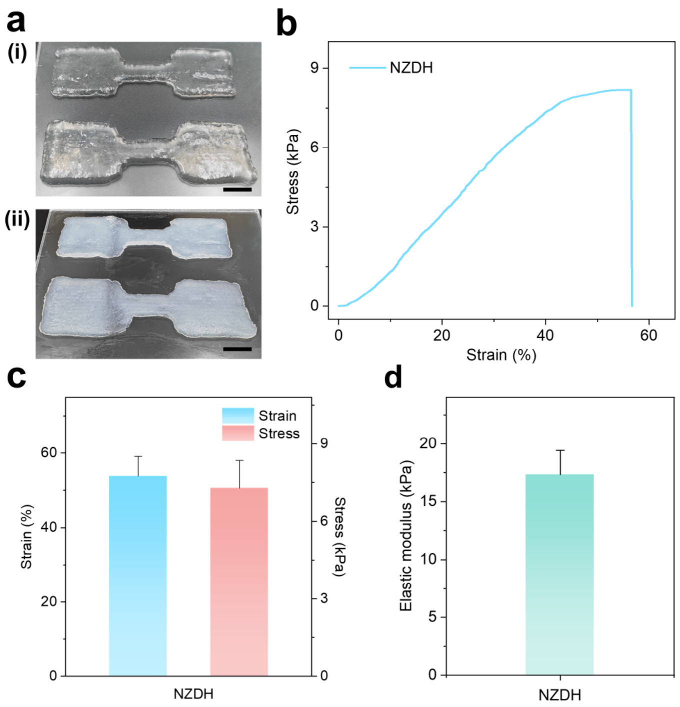


**Figure S17.** Mechanical characterizations of the NZDH interface layer. (a) Photographs of the printed hydrogel stretching mold before (i) and after curing (ii). (b-d) Stress-strain curve (b), ultimate tensile strain (c, left), fracture stress (c, right), and elastic modulus (d) of the NZDH interface layer. Scale bar, 10 mm (a).

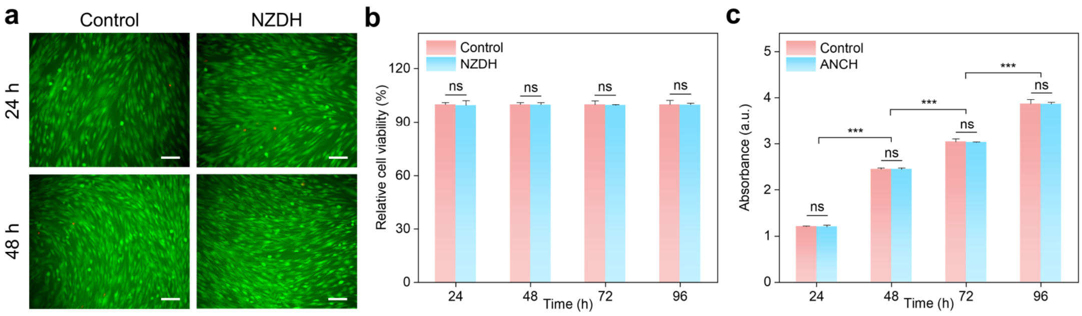


**Figure S18.** Biocompatibility assessment of fibroblasts. (a) Fluorescence micrographs of fibroblasts cultured with NZDH and control groups. (b-c) Relative cell viability (b) and proliferation (c) of fibroblasts measured by CCK-8 assays. Scale bar, 200 μm (a).

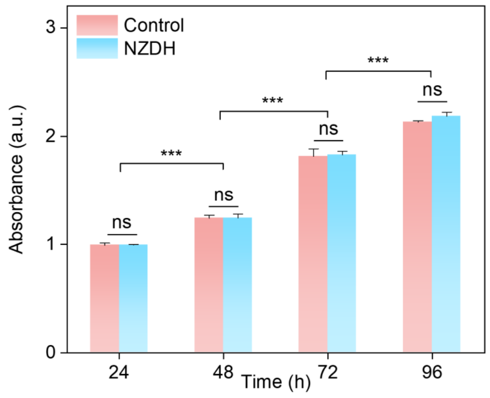


**Figure S19.** Relative cell proliferation of HaCaTs calculated via CCK-8 assays.

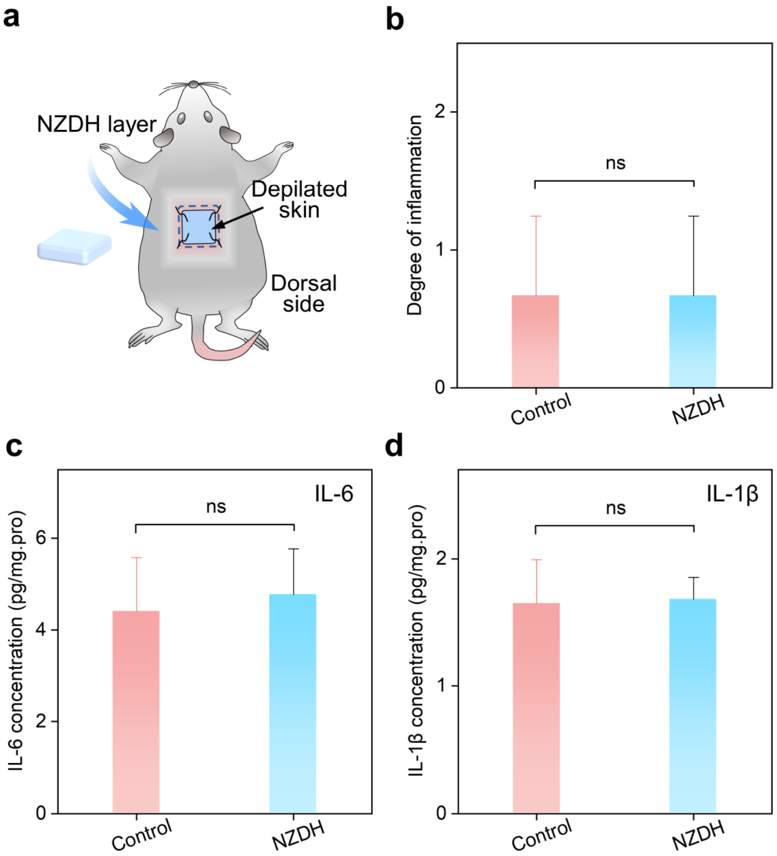


**Figure S20.** Inflammatory evaluation of NZDHs. (a) Schematic of NZDH samples sutured on the skin of SD rats. (b) Histopathological inflammation scores (0: normal; 1: minimal; 2: mild; 3: severe) assessed by the blinded pathologist after 3-day skin attachment. (c and d) Elisa assay for measurements of IL-6 (c) and IL-1β (d) levels.

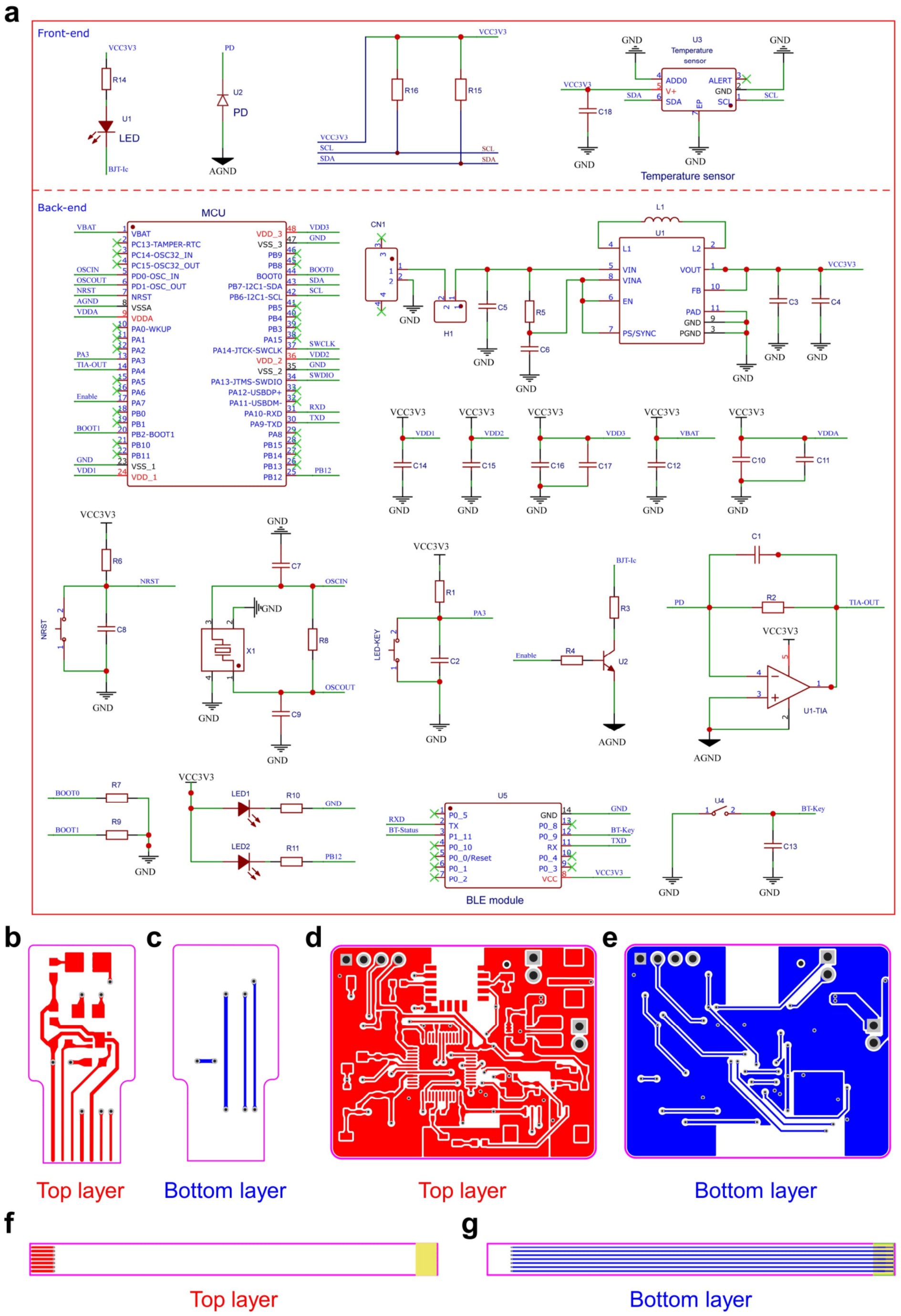


**Figure S21.** Circuit design of the hydrogel biosensor. (a) Detailed circuit diagram. (b-f) Spatial layout of the top and bottom layer of the front-end (b, c), back-end (d, e), and FFC (f, g).

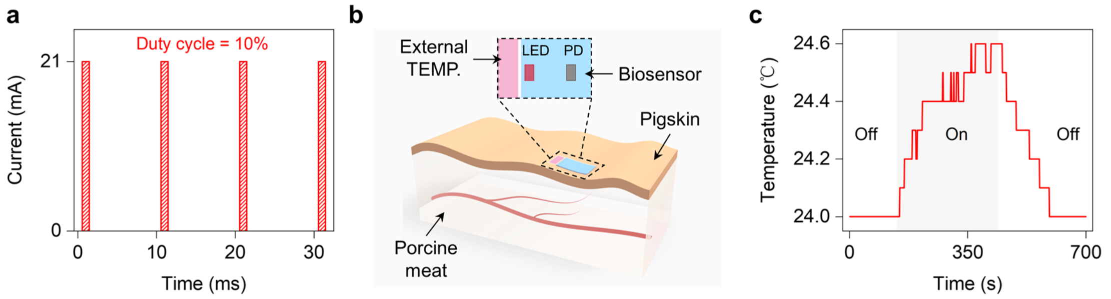


**Figure S22.** Thermal characteristics of the hydrogel biosensor. (a) Schematic illustration of 10% duty cycle of the LED. (b) Detailed measurement setup for recording temperature variations of the pigskin during LED operation. (c) Measured temperature variations of the pigskin at regions near the LED during normal operation (10% duty cycle, 21mA) of the biosensor for 5 min.

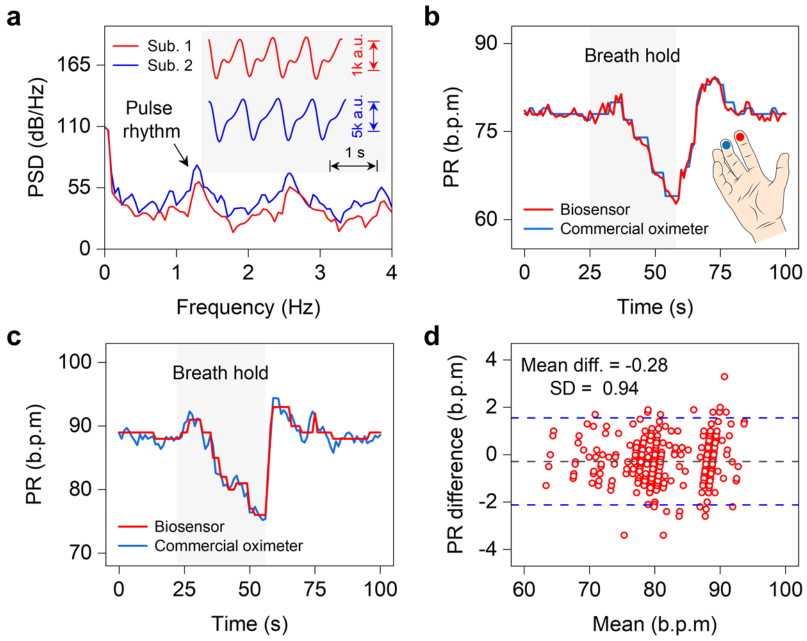


**Figure S23.** Signal acquisition capability of the hydrogel biosensor. (a) The biosensor was used to collect standard PPG waveforms from two healthy subjects (inset). These signals show clear pulse rhythms in their power spectra. PSD, power spectral density; Sub., subject. (b-c) PR measurements from two healthy subjects during a period of rest followed by a breath hold and then rest for another period using the biosensor and a commercial pulse oximeter. For data recording, the biosensor and the oximeter were attached to the index finger and the middle finger of the subject, respectively (b). It is noted that PR measurements of the biosensor are calculated from PPG signals. (d) Bland–Altman analysis of PR measurements between the biosensor and the commercial oximeter. Comparison test shows great agreement for PR. Mean difference (diff.) = -0.28 b.p.m, the standard deviation (SD) = 0.94 b.p.m.

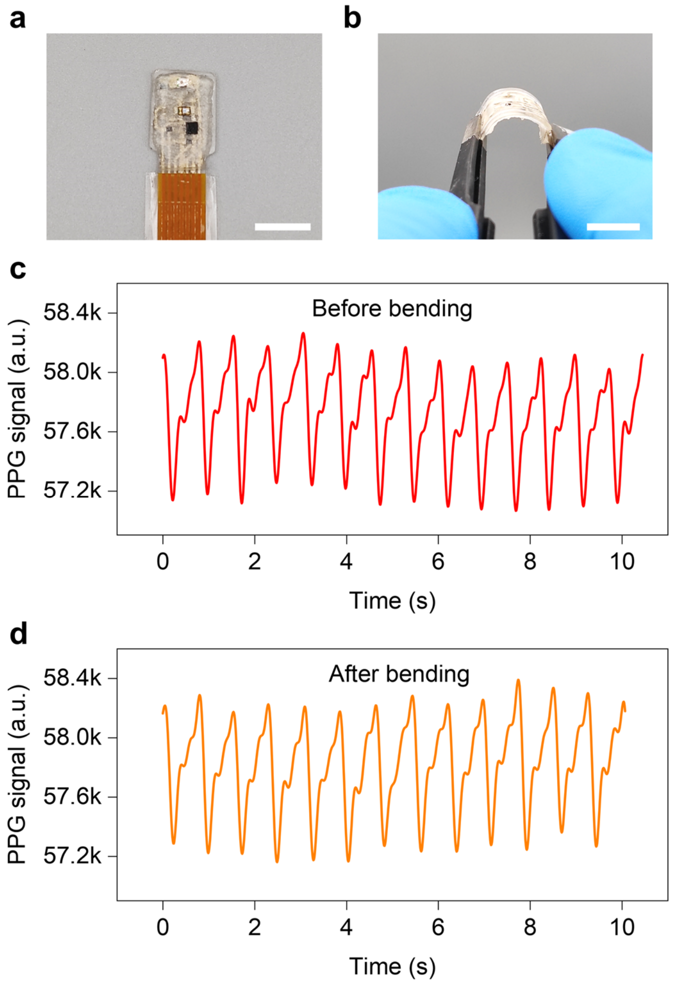


**Figure S24.** Measurement reliability of the hydrogel biosensor. (a-b) Photographs of the front-end of the biosensor before (a) and after bending (b). (c-d) PPG signals collected by the biosensor before (c) and after bending biosensor 15 times (d). Results demonstrate that the biosensor maintains stable measurement performances even after 15-time bending. a.u., arbitrary unit. Scale bars, 10 mm (a, b).

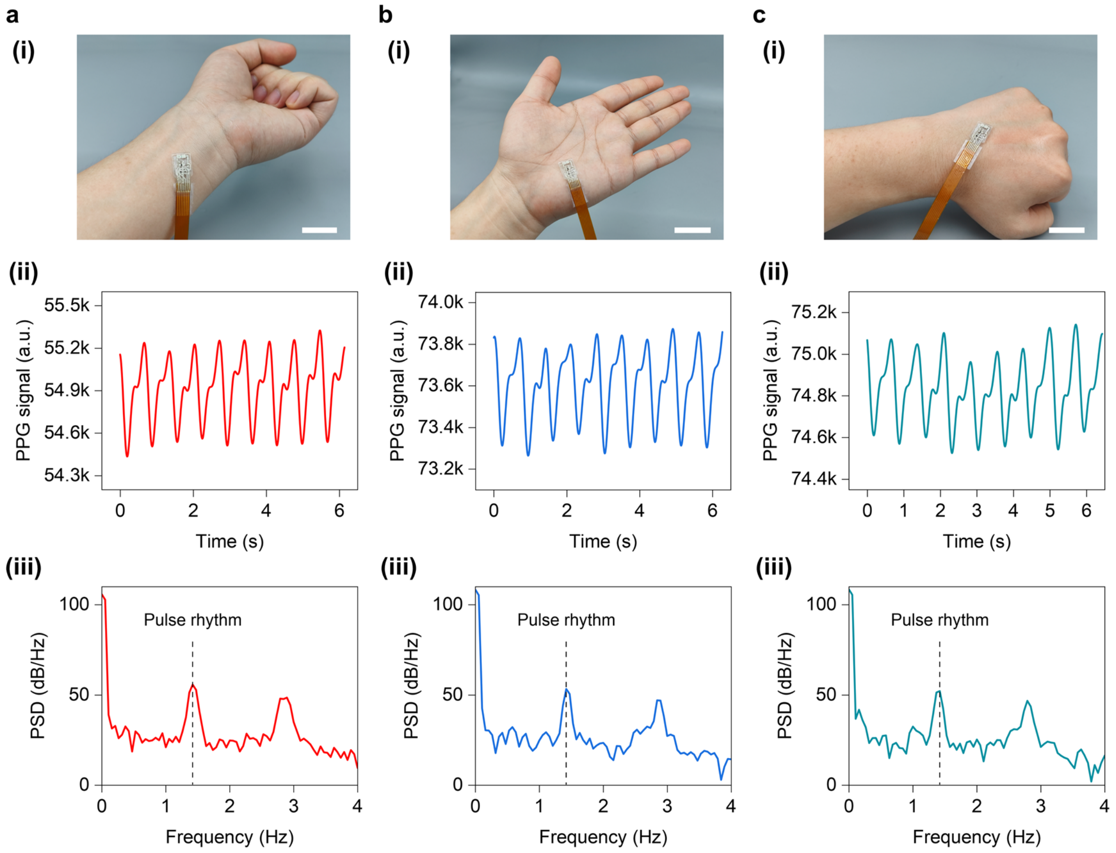


**Figure S25.** Measurements by the hydrogel biosensor on different sites of a healthy subject. (a-c) PPG signals were obtained from the wrist (a), palm (b), and back of hand (c) of a healthy subject, and all signals show clear morphological characteristics and distinct pulse rhythms. Scale bars, 2 cm (a-c).

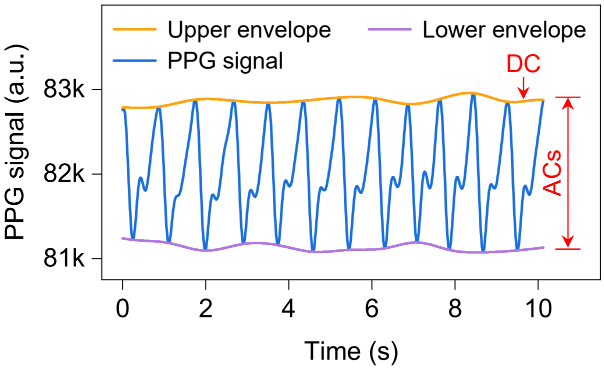


**Figure S26.** The calculation of PI. PI can be obtained by calculating the ratio between the alternating current and direct current components of PPG signals. The alternating current component is defined as the difference between the upper and lower envelopes (ACs), while the direct current component is defined as the upper envelope (DC). The calculation of PI can be expressed as: PI = ACs/DC.

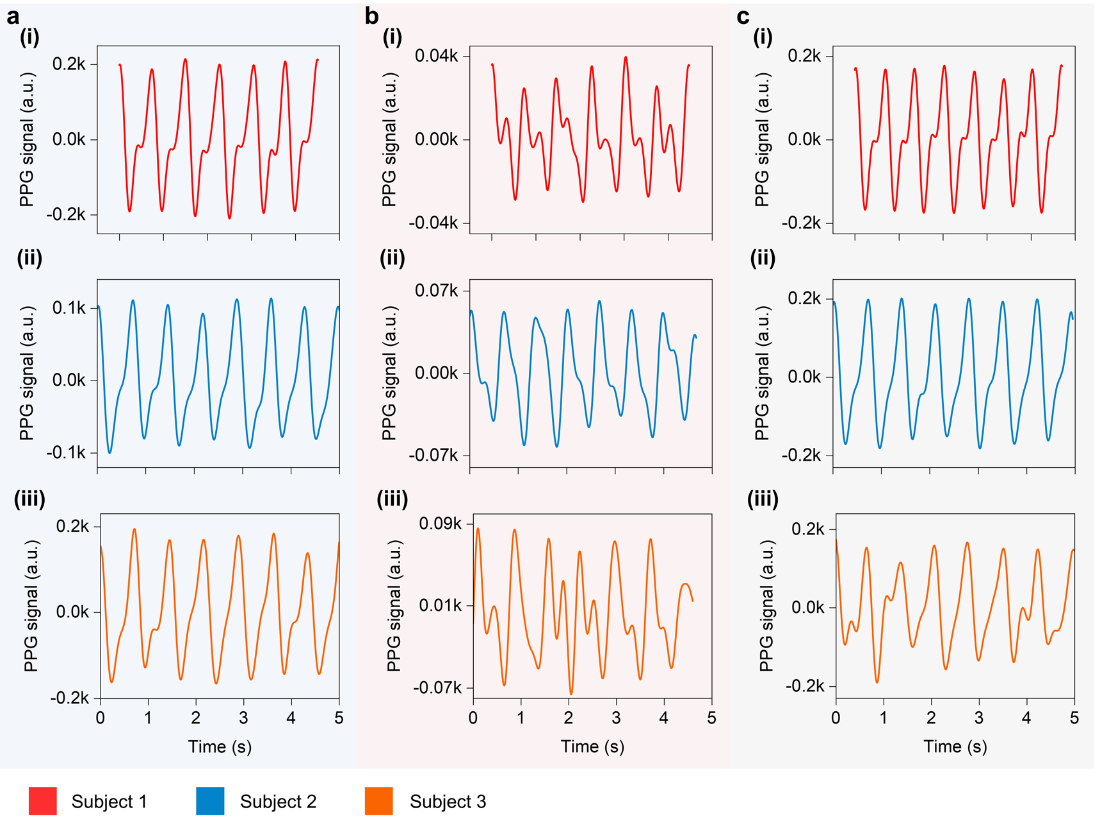


**Figure S27.** Effects of venous congestion on PPG waveforms. (a-c) Measurements of PPG waveforms from three healthy subjects under different blood circulation conditions including normal blood circulation, venous congestion, and revascularization. All PPG signals were recorded from the perforator site of the ulnar artery on the subject's wrist. Before the cuff was applied to subjects, PPG signals exhibited standard waveforms (a). When venous congestion induced by the cuff pressure occurred, PPG signals presented reverse waveforms (b). However, when the cuff pressure was released, PPG signals reverted to standard waveforms (c). These results indicate that venous congestion can transform standard PPG waveforms into reverse waveforms.

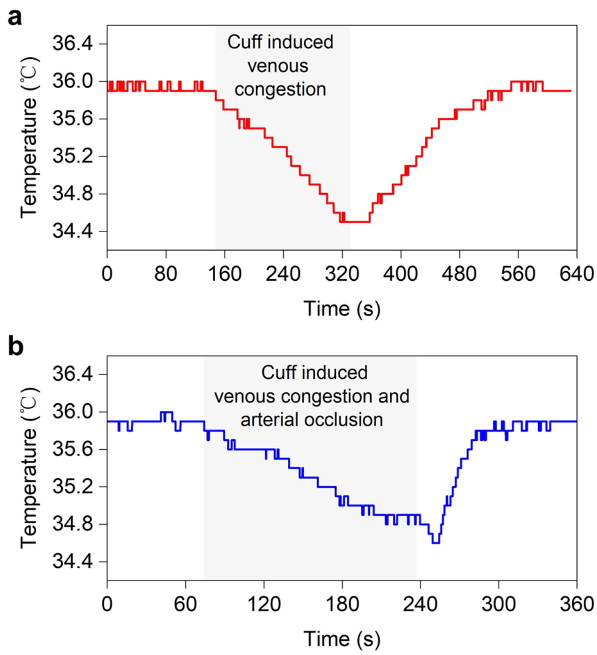


**Figure S28.** Effects of venous congestion and total occlusion on skin temperature. (a-b) Detailed changes in skin temperature of a healthy subject during the occurrence of venous congestion (a) and total occlusion (b). The venous congestion was induced by the cuff pressure of 60 mm Hg (a), and the total occlusion including venous congestion and arterial occlusion was induced by the cuff pressure of 180 mm Hg (b). Before the cuff was applied to the subjects, the skin temperature was maintained at a stable level. When venous congestion and total occlusion occurred, the skin temperature decreased. After the pressure was released, the skin temperature gradually increased to the initial level.

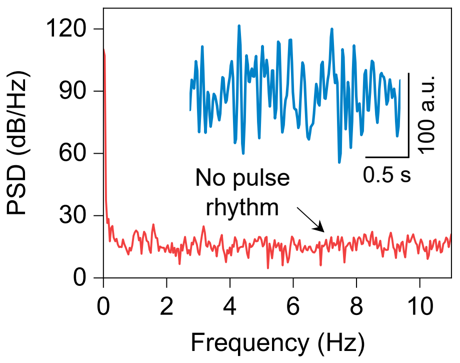


**Figure S29.** Additional data from animal tests. Representative PPG signals from stage 9 shows no pulsatile waveform (inset) or pulse rhythm.

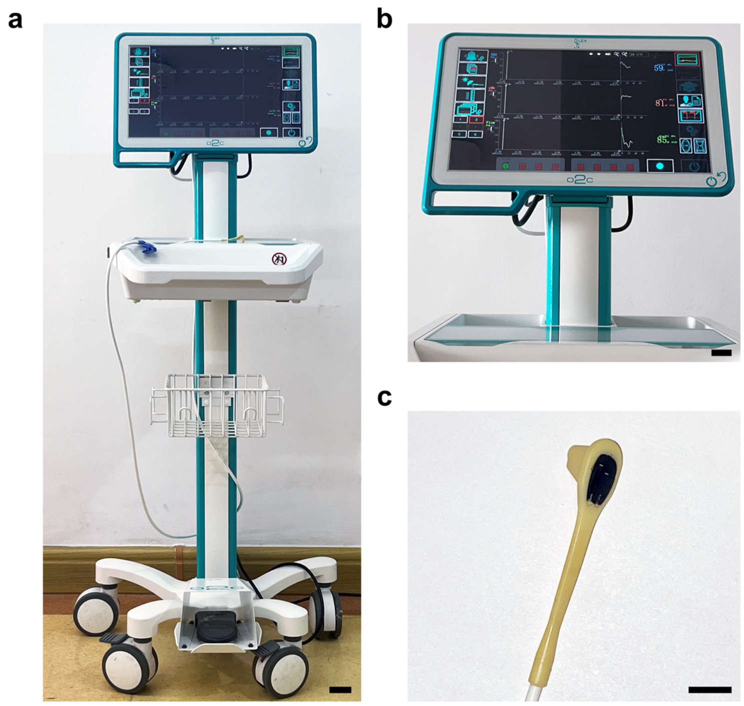

**Figure S30.** Photographs of the O2C system. (a) Photograph of the overall view of the O2C system. (b) Measurements of $S_tO_2$, Flow, and rHb can be displayed on the O2C screen. (c) Photograph of the O2C probe. Scale bars, 4 cm (a), 2 cm (b), 1 cm (c).

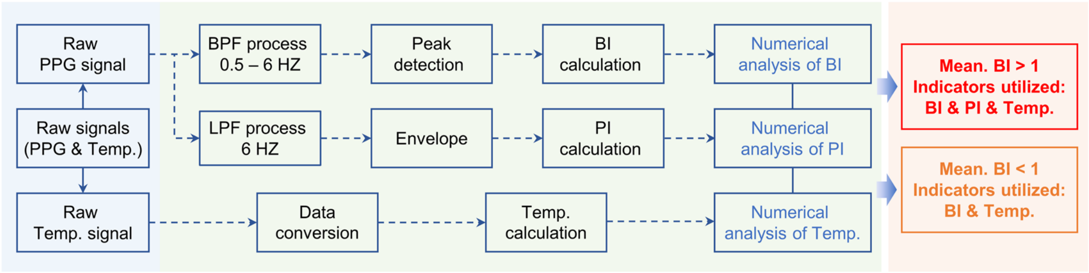


**Figure S31.** Flowchart of the data analysis procedure of the hydrogel biosensor. During data analysis procedure, BI, PI, and temperature can be calculated to monitor blood circulation of free flaps. Notably, when the calculated mean value of BI (Mean. BI) is less than 1, PI will not be utilized for analyzing blood circulation status of the flap. Temp., temperature; BPF, bandpass filtering; LPF, lowpass filtering.

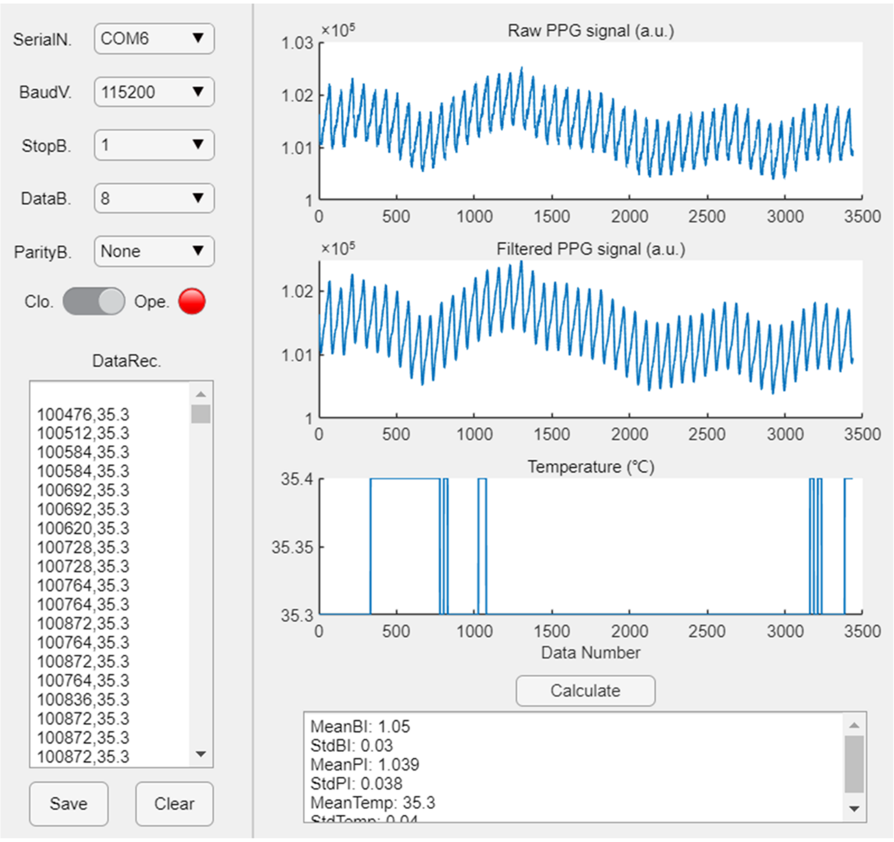


**Figure S32.** The customized GUI for signal display and analysis. Raw PPG signal, filtered PPG signal, and temperature signal can be displayed in the GUI. BI, PI, and skin temperature can be calculated from these signals.

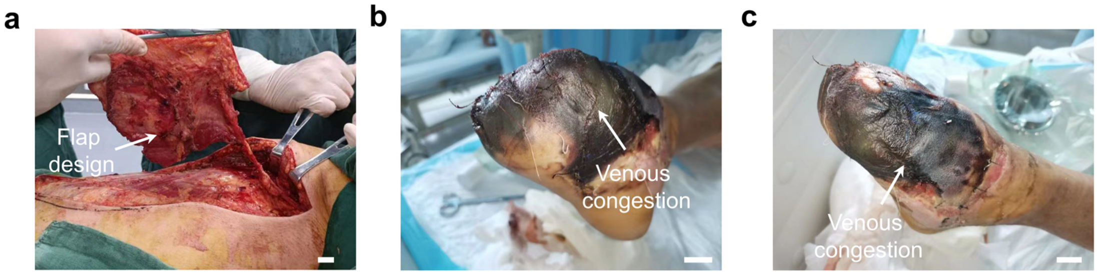


**Figure S33.** Photographs from case 1. A LD free flap was chosen for tissue coverage, with the anterior tibial artery and its accompanying vein anastomosed. (a) Photographs show that the flap was elevated from the latissimus dorsi muscle. (b-c) Photographs show the purple appearance of the flap, indicating venous congestion. Scale bars, 10 mm (a-c).

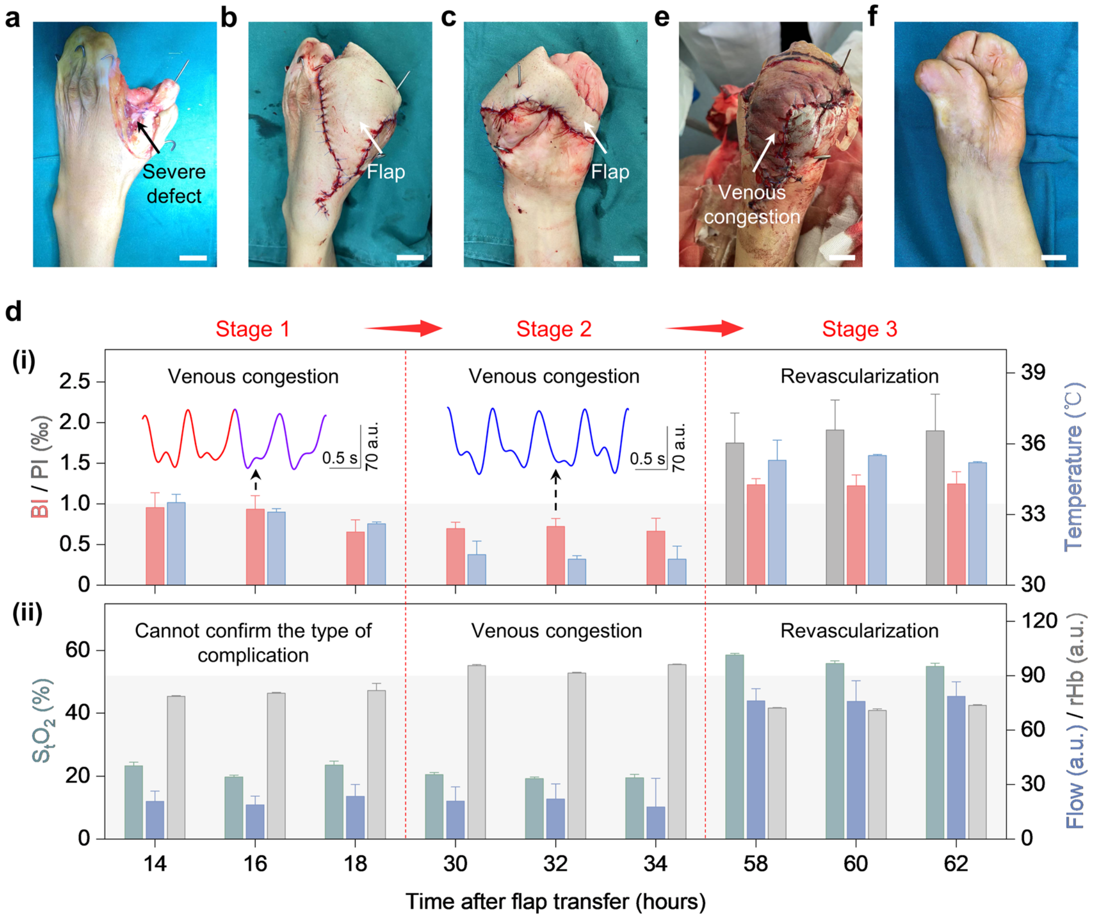


**Figure S34.** Monitoring the anterolateral thigh free flap in case 2. An ALT free flap was transferred for treating burn scars. The radial artery and its accompanying vein, along with a cephalic vein, were anastomosed. In stage 1, each group of PPG signals contained both standard and reverse PPG waveforms, which led to BI being slightly less than 1 and the speculation that mild venous congestion might occur. Meanwhile, relatively low values of StO2 (~ 23%) and Flow (~ 20 a.u.) but relatively high value of rHb (~82 a.u.) measured by O2C merely indicated a potential blood circulation complication, but were unable to predict the specific type of the complication. In stage 2, all PPG signals transformed into reverse waveforms, and BI became much less than 1. In this stage, the rHb value from O2C increased above 90 a.u.. In stage 3, the BI rose apparently above 1, with both PI and temperature increasing gradually. Meanwhile, the rHb value decreased below 90 a.u. (~72 a.u.), and $S_tO_2$ and Flow also increased to high levels ($S_tO2$ > 50%, Flow > 60 a.u.). (a) Photograph of patient's left hand with severe burn scars. (b-c) Photographs of the flap transferred after surgery. (d) Measurement results by the biosensor and O2C (mean ± SD, n > 30 per group). (e) Photograph of the flap with purple skin color. (f) Photograph shows that the flap survived ultimately. Scale bars, 10 mm (a-c, e, f).

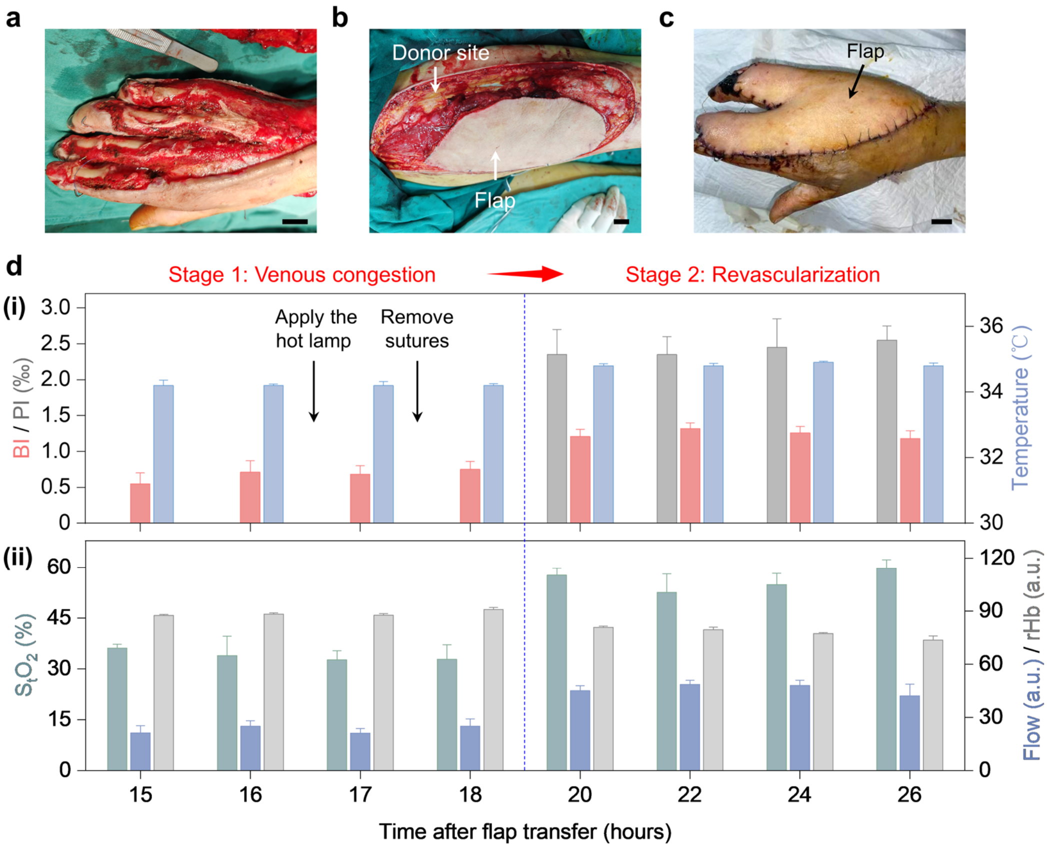


**Figure S35.** Monitoring the anterolateral thigh free flap in case 3. An ALT free flap was transferred to the dorsum of the right hand of a 40-year-old woman to cover the soft tissue defect. Intraoperatively, the radial artery and its accompanying vein, along with the cephalic vein, were anastomosed to an artery and two veins of the flap, respectively. In stage 1, the measurements of the biosensor (BI < 1) and O2C (High rHb ~90 a.u.) both indicated venous congestion of the flap. At 16 hours after transfer, the surgeon also suspected a high risk of venous congestion and applied a hot lamp for approximately 30 minutes. In addition, the surgeon removed sutures of the flap at 17 hours after transfer to relieve external pressures. In stage 2, the surgeon confirmed the improved blood circulation of the flap, which was consistent with the measurements of the biosensor and O2C. The flap survived eventually. (a) Photograph of the patient's right hand with soft tissue defect. (b) Photograph shows that the flap was designed and harvested from the anterolateral area of the left thigh. (c) Photograph of the surviving flap at 48 hours after transfer. (d) Measurement results by the biosensor (i) and O2C (ii) (mean ± SD, n > 30 per group). Scale bars, 10 mm (a-c).

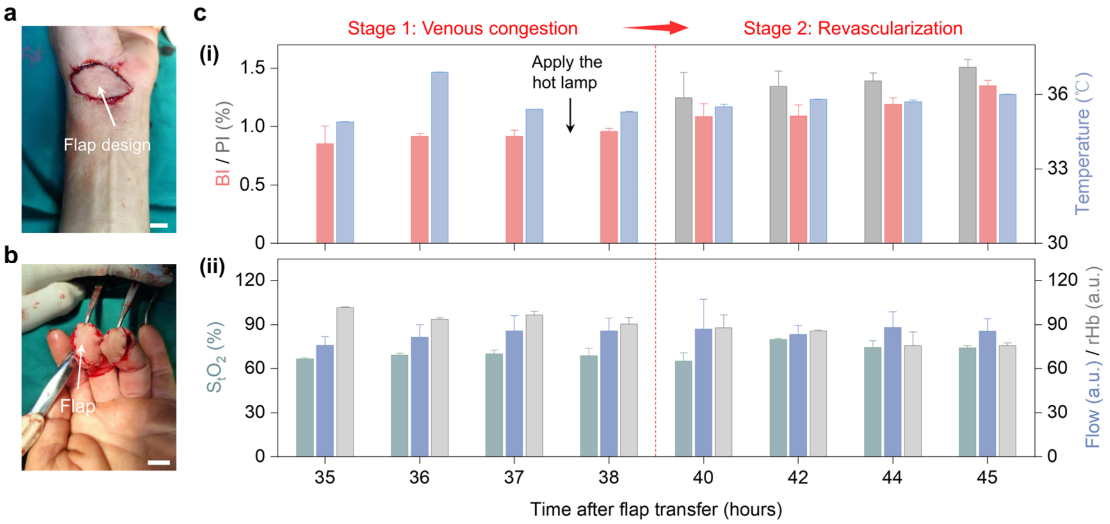


**Figure S36.** Monitoring the free flap from the wrist in case 4. A 34-year-old man lost the tip of his left middle finger. To address this injury, a free flap was designed from the flexor aspect of his left wrist and transferred to cover the defect. During the surgery, a digital artery and its accompanying vein were anastomosed to an artery and a vein of the flap, respectively. In stage 1, the measurements of the biosensor (BI < 1) and O2C (rHb > 90 a.u.) indicated the occurrence of the venous congestion. At 37 hours after transfer, the surgeon confirmed the venous congestion and applied a hot lamp for approximately 25 minutes. In stage 2, symptoms of the venous congestion were effectively relieved, which was verified by clinical assessment and measurements of the biosensor and O2C. Ultimately, this flap survived successfully. (a-b) Photographs show that the flap was harvested from the left wrist (a), and transferred to the left middle finger (b). (c) Measurement results by the biosensor (i) and O2C (ii) (mean ± SD, n > 30 per group). Scale bars, 10 mm (a, b).

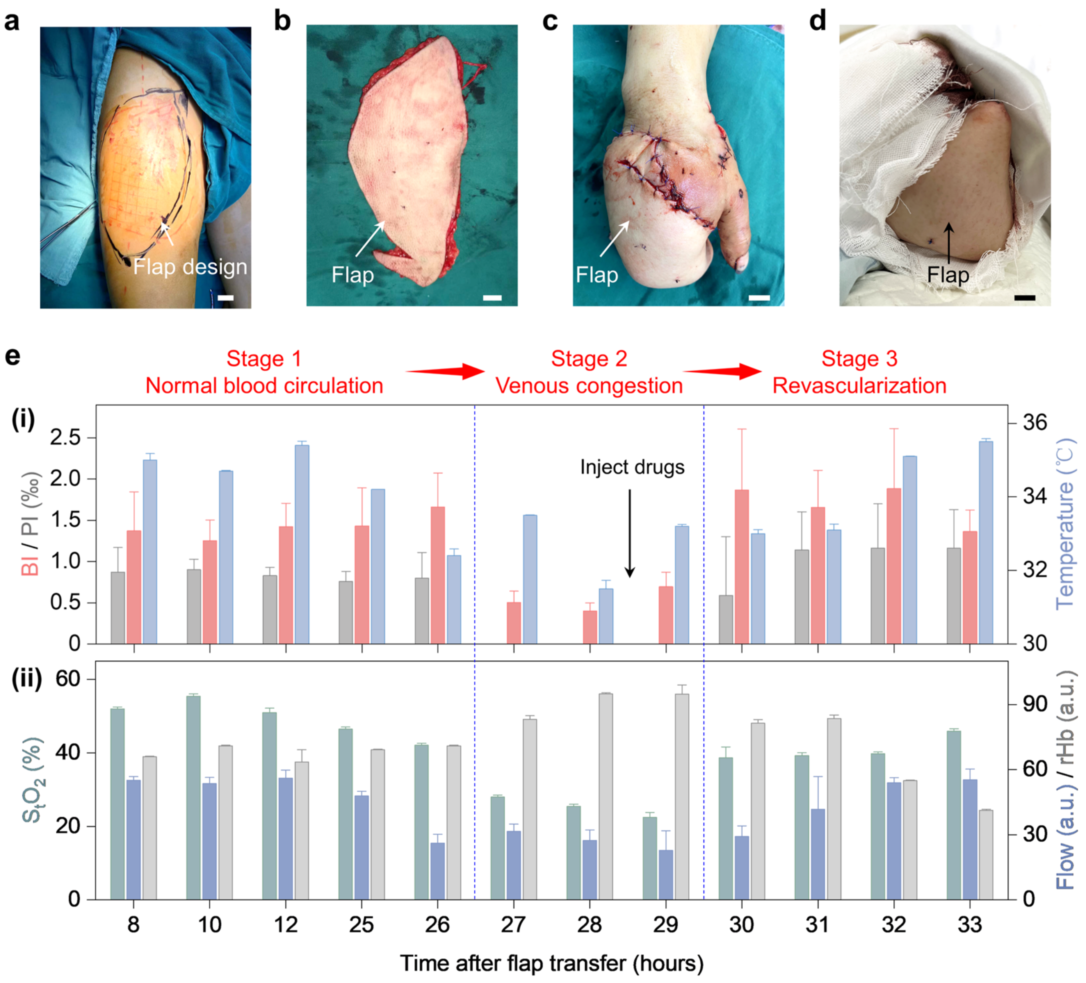


**Figure S37.** Monitoring the anterolateral thigh free flap in case 5. A 37-year-old man suffered severe soft tissue defect of his right hand, and the ALT free flap from his right leg was designed for flap transfer. Two digital arteries and a hand dorsal vein, as well as the cephalic vein, were anastomosed to two arteries and two veins of the flap, respectively. In stage 2, short-term venous congestion was identified by both the biosensor and O2C. In particular, this blood circulation complication was also confirmed by the surgeon at 28 hours after transfer, and papaverine was injected subsequently by the surgeon to promote blood circulation. In stage 3, the blood circulation of the flap recovered to a normal state, and the flap survived eventually. (a-c) Photographs show that the flap was designed (a), harvested (b), and transferred to the right hand (c). (d) Photograph of the surviving flap at 48 hours after transfer. (e) Measurement results by the biosensor (i) and O2C (ii) (mean ± SD, n >30 per group). Scale bars, 10 mm (a-d).

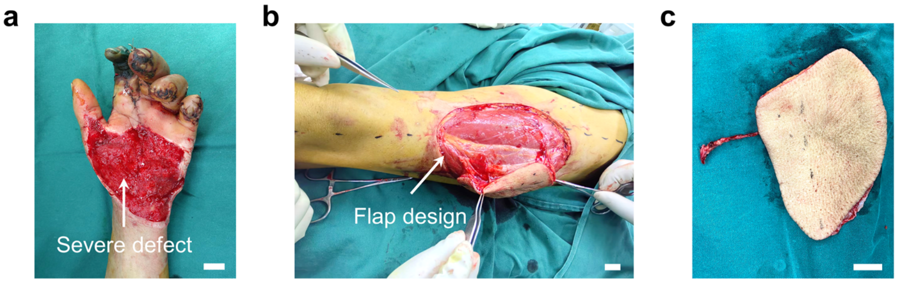


**Figure S38.** Photographs from case 6. (a) Photograph shows the patient's left hand with soft tissue defect. (b-c) Photographs show that the flap was elevated (b) and harvested (c) from the anterolateral area of the left thigh. Scale bars, 10 mm (a-c).

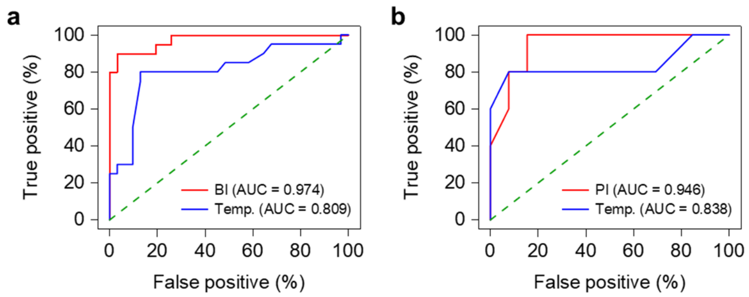


**Figure S39.** ROC curves for the detection of venous congestion and arterial spasm. (a) ROC curves of BI and temperature for detecting venous congestion. (b) ROC curves of PI and temperature for detecting arterial spasm. Temp., temperature.

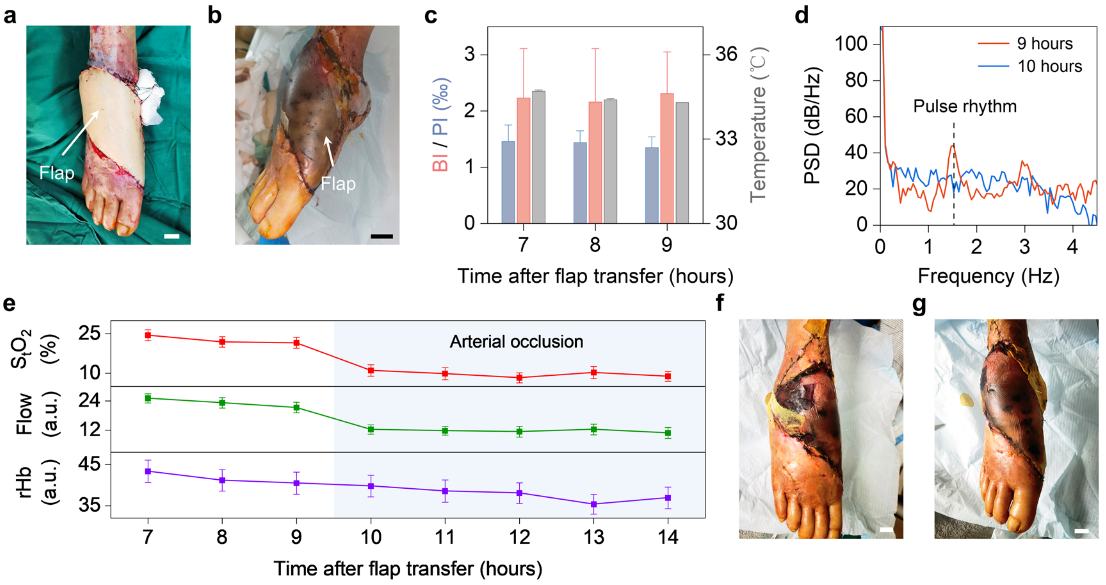


**Figure S40.** Monitoring the anterolateral thigh free flap in case 7. (a) Photograph shows that the ALT free flap was transferred to the patient's foot. (b) Photograph of the flap with dark skin color. (c) Measurement results by the biosensor (mean ± SD, n > 30 per group). (d) Power spectrum shows regular pulse rhythm for PPG signals at 9 hours after transfer but loss of pulse rhythm for PPG signals at 10 hours after transfer. (e) Measurement results by the O2C (mean ± SD, n > 30 per group). (f-g) Photographs show reduction in the color depth of the dark appearance of the flap, indicating the alleviation of the arterial occlusion. Scale bars, 10 mm (a, b, f, g).

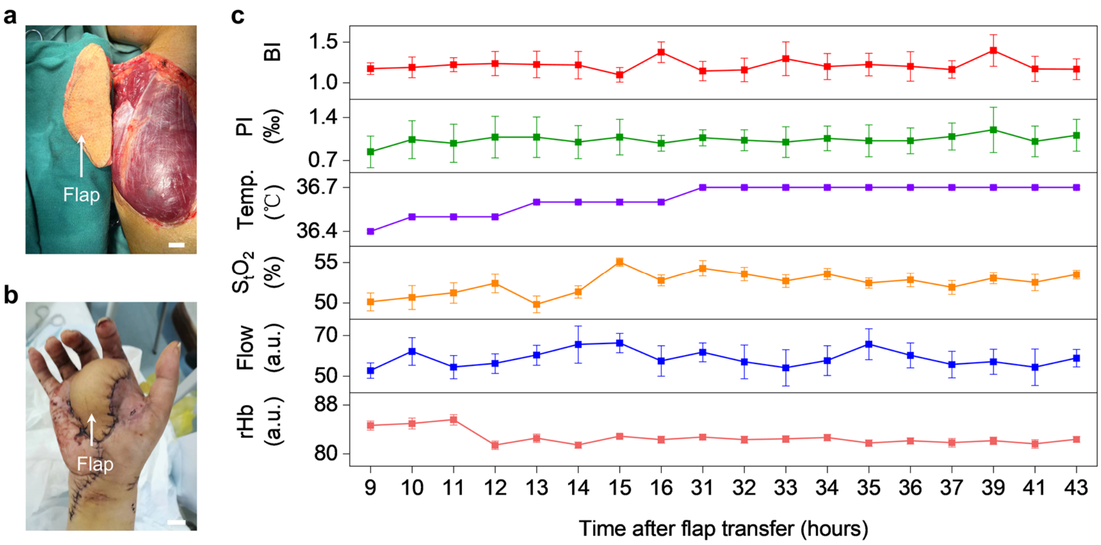


**Figure S41.** Monitoring the medial sural artery perforator free flap in case 8. A 34-year-old man suffered soft tissue necrosis on the radial side of his right palm. The medial sural artery perforator (MSAP) flap from the left leg was designed for flap transfer. The radial artery and its accompanying vein, as well as the superficial vein, were anastomosed to an artery and two veins of the flap, respectively. Postoperative blood circulation monitoring was performed from 9 hours to 43 hours after transfer. During the monitoring process, BI was consistently above 1 and there was no clear drop in PI or temperature, and all indicators of O2C remained within the normal range. Meanwhile, based on the clinical assessment, the surgeon also confirmed the normal blood circulation of the flap. The flap survived eventually. (a) Photograph of the flap elevation. (b) Photograph of the right palm at 48 hours after flap transfer, showing no blood circulation complication. (c) Measurement results by the biosensor and O2C (mean ± SD, n >30 per group). Scale bars, 10 mm (a, b).

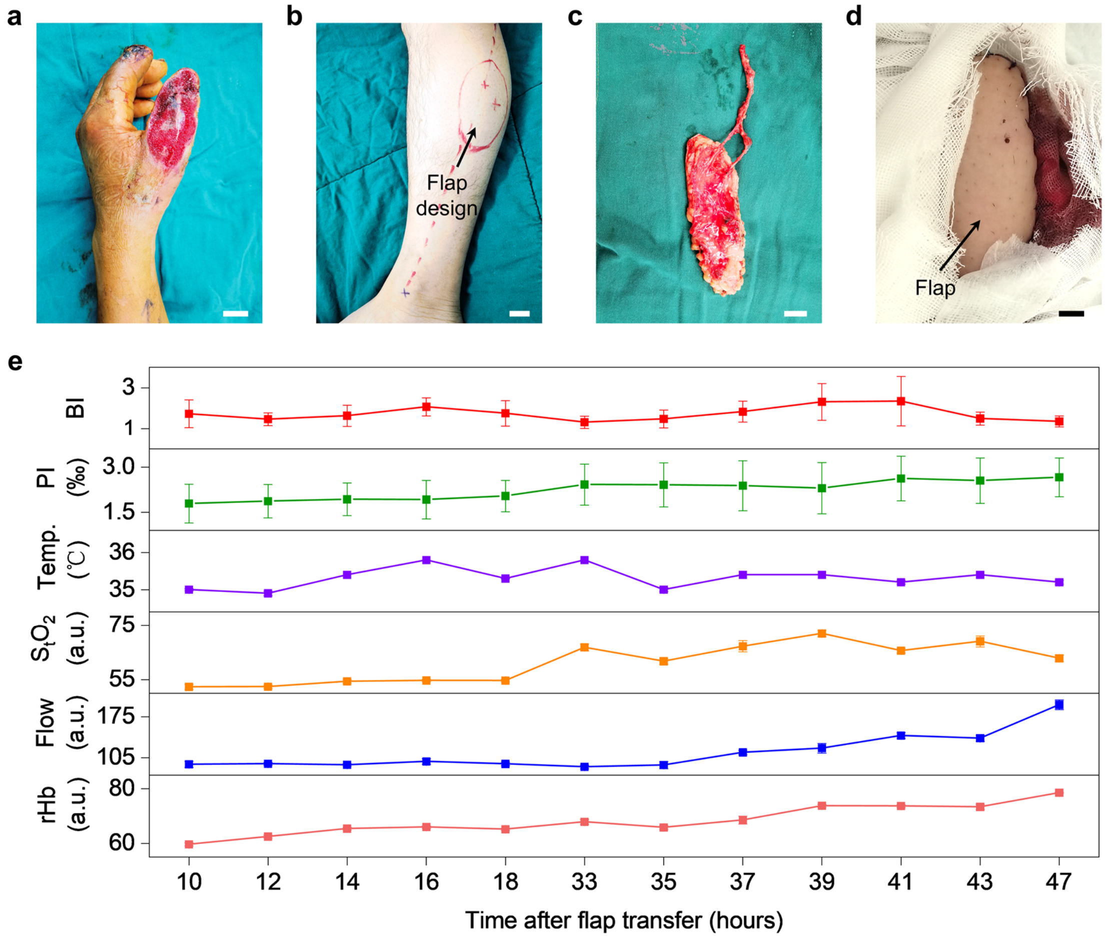


**Figure S42.** Monitoring the medial sural artery perforator free flap in case 9. A 28-year-old man suffered open injury in his left thumb, and a MSAP free flap was transferred for wound coverage. Intraoperatively, the radial artery and its accompanying vein, as well as the cephalic vein, were anastomosed to an artery and two veins of the flap, respectively. Due to the stable blood circulation postoperatively, the flap survived ultimately. (a) Photograph illustrates the patient's left thumb with soft tissue defect. (b-d) Photographs show that the MSAP flap was designed (b), harvested (c), and transferred to the left thumb (d). (e) Measurement results by the biosensor and O2C (mean ± SD, n >30 per group). Scale bars, 10 mm (a-d).

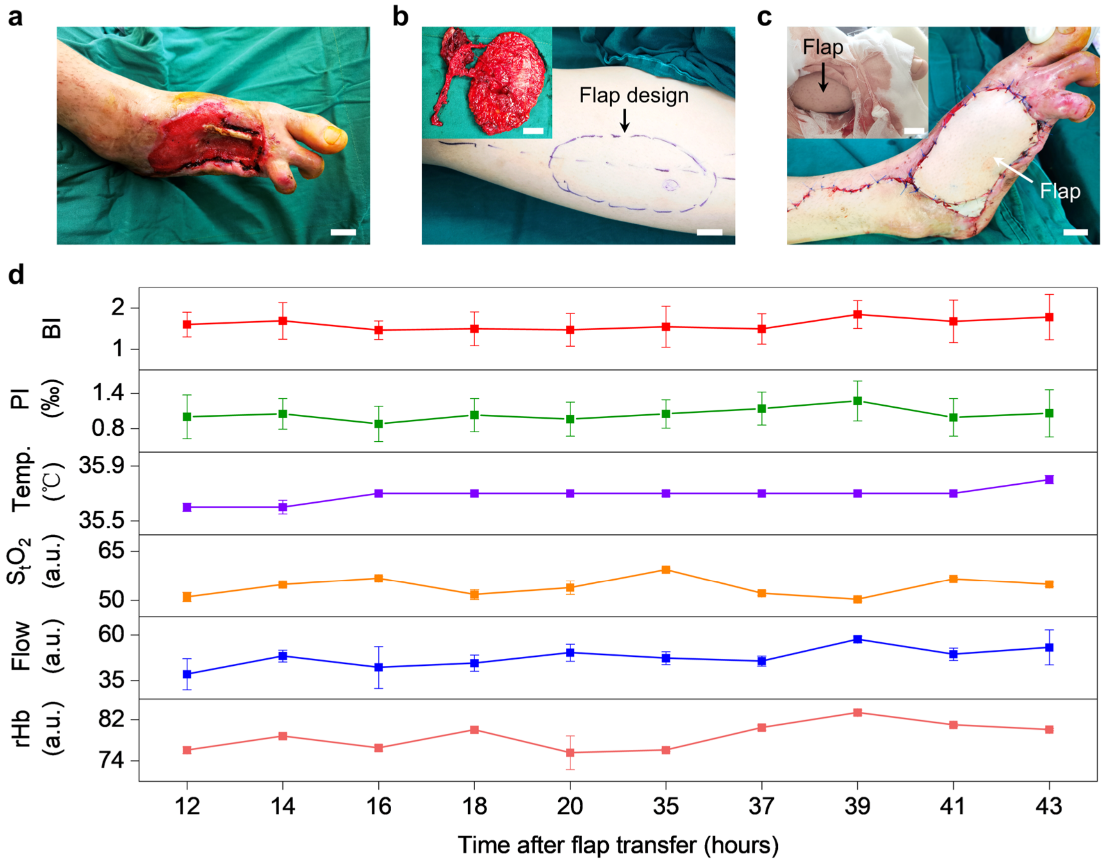


**Figure S43.** Monitoring the anterolateral thigh free flap in case 10. A 40-year-old man suffered severe metatarsal bone exposure of his right foot. An ALT free flap from his left leg was transferred for tissue coverage, with the anterior tibial artery and its accompanying vein being anastomosed to an artery and a vein of the flap, respectively. Clinical assessment and measurements of the biosensor and O2C all indicated that there was no blood circulation complication from 12 hours to 43 hours after flap transfer. The flap survived eventually. (a) Photograph of the patient's right foot with soft tissue defect. (b) Photograph of the flap design, and the ALT flap harvested (inset). (c) The flap was transferred to the foot during the surgery. Inset shows the transferred flap with normal color at 48 hours after the surgery, indicating no blood circulation complication. (d) Measurement results by the biosensor and O2C (mean ± SD, n >30 per group). Scale bars, 10 mm (a-c).

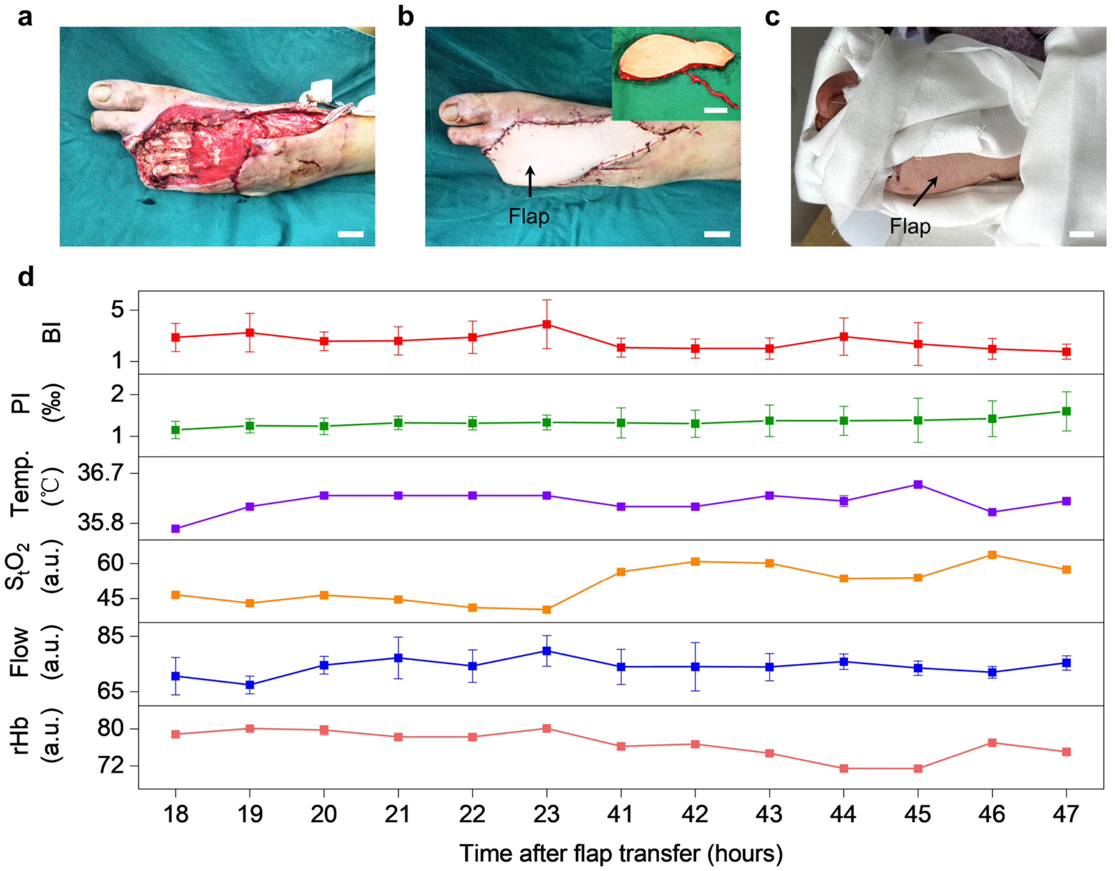


**Figure S44.** Monitoring the anterolateral thigh free flap in case 11. An ALT free flap was transferred to the left foot of a 20-year-old woman to cover the severe soft tissue defect. During the surgery, the anterior tibial artery and its accompanying vein were anastomosed to an artery and a vein of the flap, respectively. No blood circulation complication was detected during continuous blood circulation monitoring from 18 hours to 47 hours after transfer, and the flap survived eventually. (a) Photograph presents the patient's left foot with severe soft tissue defect. (b) Photographs show that the ALT flap was harvested (inset), and transferred to the left foot. (c) Photograph of the surviving flap at 48 hours after transfer. (d) Measurement results by the biosensor and O2C (mean ± SD, n >30 per group). Scale bars, 10 mm (a-c).

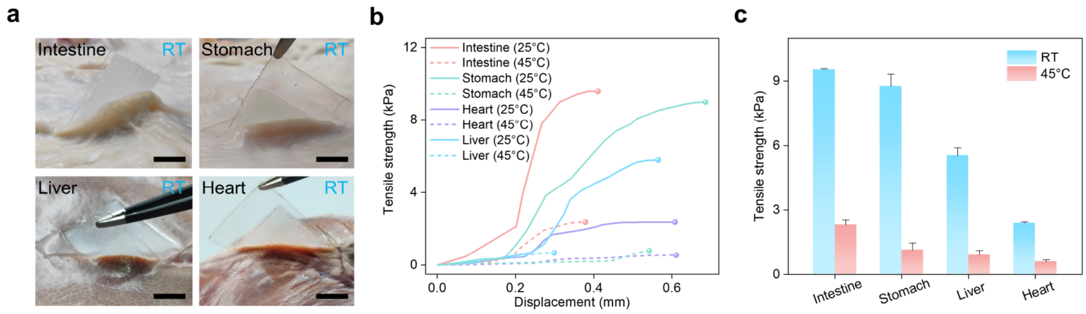


**Figure S45.** Adhesive properties of hydrogel interface layers on different porcine tissues. (a) Photographs of tight integration between the hydrogel and porcine tissues at RT. (b-c) Tensile strength-displacement curves (b) and corresponding strength values (c) of NZDHs when adhered to different porcine tissues (*e.g.*, intestine, stomach, liver, and heart) at RT and 45°C, respectively. Scale bar, 10 mm (a).

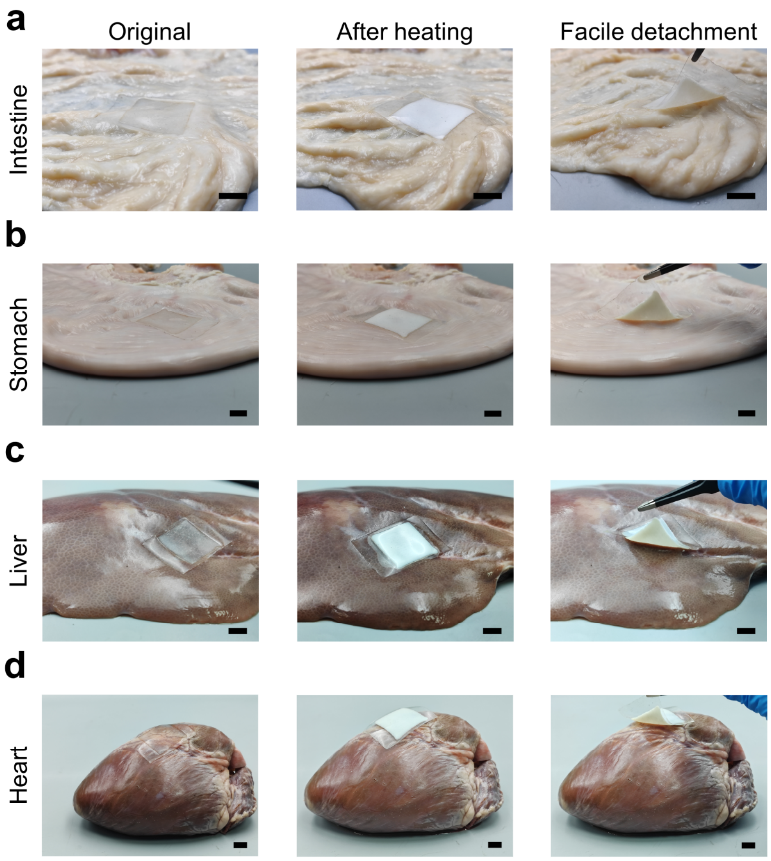


**Figure S46.** Comparisons of thermoresponsive NZDHs when adhered to different porcine issues. (a-d) Images of NZDHs achieving seamless integration at RT and benign detachment after heating on the intestine (a), stomach (b), liver (c), and heart (d), respectively. Scale bars, 10 mm (a-d).

**Table S1. Comparisons of techniques for blood circulation monitoring.**

| Method | Accuracy | Rapidly Responsive | Easy to Interpret | Easy to Operate | Cost (US Dollars) | Ref. |
|---|---|---|---|---|---|---|
| Acoustic Doppler sonography (ADS) | Medium, cannot differentiate different blood circulation complications | + | - | + | ~$1300 | (*1-3*) |
| Color Doppler ultrasonography (CDU) | High | + | - | - | $30,000 to $225,000 | (*1, 4*) |
| Laser Doppler flowmetry (LDF) | Medium, cannot differentiate different blood circulation complications | + | + | + | ~$4670 | (*5, 6*) |
| O2C (LDF + tissue spectrophotometry) | High | + | + | + | $78, 700 | (*7*) |
| Near-infrared spectroscopy (NIRS) sensor | Medium, results show poor reproducibility | + | + | + | ~$40000 | (*5, 8, 9*) |
| Cuffless blood pressure monitoring | Medium, cannot differentiate different blood circulation complications | + | + | + | / | (*10*) |
| Clinical assessment | / | - | - | + | / | / |
| **Biosensor** | **High** | **+** | **+** | **+** | **$18.50** | **This work** |

**Table S2. Comparisons of hydrogels as interface layers for bioelectronics.**

| Materials | Methods | Patterning precision | Manufacturing time | Adhesion strength | Tunable adhesion | Application | Ref. |
|---|---|---|---|---|---|---|---|
| PNIPAM/PAM/PEDOT:PSS | Mold pouring | / | / | ~150 J $m^{-2}$ | Yes | Wound care | *(11)* |
| AHBH | Mold pouring | >5 mm | >5 min | 59.7 N $m^{-1}$ | No | Electrode | *(12)* |
| CNF-DA/PAA | Mold pouring | / | >5 min | 89.13 J $m^{-2}$ | Yes (15-fold) | TENG | *(13)* |
| AAm-Alg/Chitosan | Mold pouring | / | / | ~1750 J $m^{-2}$ | No | Bioadhesives | *(14)* |
| PVA/PAA-S-S-NHS | Mold pouring | / | / | ~270 J $m^{-2}$ | Yes (8-fold) | Electrodes | *(15)* |
| NIH | Mold pouring | >1 mm | >20 min | >4 kPa | No | Electrodes | *(16)* |
| PAAM/PAA | Mold pouring | / | / | ~200 J $m^{-2}$ | Yes (6-fold) | Bioadhesives | *(17)* |
| QDM-Alg | Mold pouring | >5 mm | >10 min | 20 kPa | Yes (4-fold) | Tissue sealing | *(18)* |
| PDEH | Mold pouring | >3 mm | >5 min | 29.2 kPa | Yes (7.8-fold) | Electrodes | *(19)* |
| PBAc-NHS/SA | Mold pouring | / | / | ~300 J $m^{-2}$ | Yes (10-fold) | Bioadhesives | *(20)* |
| GCMCS | Mold pouring | / | / | / | No | Wound Healing | *(21)* |
| DGMH | Mold pouring | >2 mm | >15 min | 26.1 kPa | Yes (7-fold) | Perforating artery locating | *(22)* |
| **NZDH** | **Direct printing** | **720 µm** | **<30 s** | **27.8 kPa** | **Yes (10.8-fold)** | **Detection of blood circulation complication** | **This work** |

**Table S3. Summary of representative clinical cases.**

| Case number | Gender | Type of the flap | Injured site | Detection of blood circulation complications | | |
|---|---|---|---|---|---|---|
| | | | | Biosensor | O2C | Clinical assessment |
| 1 | Male | LD free flap | Left foot | Venous congestion | Venous congestion | Venous congestion, later than biosensor |
| 2 | Male | ALT free flap | Left hand | Venous congestion | Venous congestion, later than biosensor | Venous congestion, later than biosensor |
| 3 | Female | ALT free flap | Right hand | Venous congestion | Venous congestion | Venous congestion, later than biosensor |
| 4 | Male | Free flap from the wrist | Left middle finger | Venous congestion | Venous congestion | Venous congestion, later than biosensor |
| 5 | Male | ALT free flap | Right hand | Venous congestion | Venous congestion | Venous congestion, later than biosensor |
| 6 | Male | ALT free flap | Left hand | Arterial spasm | Arterial spasm | Arterial spasm |
| 7 | Female | ALT free flap | Right foot | Arterial occlusion | Arterial occlusion | Arterial occlusion, later than biosensor |
| 8 | Male | MSAP free flap | Right hand | No complication | No complication | No complication |
| 9 | Male | MSAP free flap | Left thumb | No complication | No complication | No complication |
| 10 | Male | ALT free flap | Right foot | No complication | No complication | No complication |
| 11 | Female | ALT free flap | Left foot | No complication | No complication | No complication |

**Table S4. Estimated costs of the hydrogel biosensor developed in this study.**

| Component | Unit Price | Quantity | Total |
|---|---|---|---|
| Capacitor | $0.001 | 18 | $0.018 |
| Resistor | $0.001 | 16 | $0.016 |
| Inductor | $0.004 | 1 | $0.004 |
| Buck-Boost chip | $1.5 | 1 | $1.5 |
| BJT | $0.01 | 1 | $0.01 |
| Microcontroller | $1.2 | 1 | $1.2 |
| Tact switch | $0.12 | 1 | $0.12 |
| Pin header | $0.01 | 3 | $0.03 |
| Needle stand | $0.04 | 1 | $0.04 |
| LED | $0.5 | 4 | $2 |
| PD | $0.75 | 1 | $0.75 |
| Temperature sensor | $1.4 | 1 | $1.4 |
| BLE module | $3 | 2 | $6 |
| OPA | $1.1 | 1 | $1.1 |
| Crystal | $0.1 | 1 | $0.1 |
| FFC | $0.1 | 1 | $0.1 |
| FFC connector | $0.1 | 1 | $0.1 |
| Circuit board | $2.5 | 1 | $2.5 |
| PDMS | $0.169/g | 2 g | $0.338 |
| Silver powder | $1.8/g | 0.1 g | $0.18 |
| NZDH interface layer | $0.24/per device | 1 | $0.24 |
| Printing nozzle | $0.1/per device | 1 | $0.1 |
| Syringe | $0.15/per device | 1 | $0.15 |
| Printing fabrication cost | $0.5/per device | 1 | $0.5 |
| **Total** | **$18.50 per device** | | |

BJT, Bipolar junction transistor; OPA, Operational amplifiers.

**Table S5.** Comparison of the biosensor with previously reported on-skin PPG sensors

| Sensor | Key optoelectronics | Indicators for monitoring | Self-adhesion (probe) | Tunable adhesion (probe) | Wireless transmission | Ref. |
|---|---|---|---|---|---|---|
| Multispatial PPG sensor | Eight LEDs and a PD | MW delay, PR | - | - | + | (*23*) |
| On-skin pulse oximetry sensor | Two LEDs and a PD | $SpO_2$, PR | + | - | + | (*24*) |
| Optoelectronic PPG patch | Two LEDs and a PD | $SpO_2$, PR | - | - | - | (*25*) |
| Quantum dot-based pulse oximetry sensor | Two LEDs and a PD | $SpO_2$, PR | - | - | - | (*26*) |
| Perovskite-based PPG sensor | A LED and a PD | PR | - | - | - | (*27*) |
| **Biosensor** | **A LED and a PD** | **PI, BI, PR** | + | + | + | **This work** |

MW delay, arteriolar pulse transit time; $SpO_2$, peripheral oxygen saturation.

## SI References


1. Kohlert S, Quimby AE, Saman M *et al.* Postoperative free-flap monitoring techniques. *Semin Plast Surg* 2019; **33**: 13-6.
2. Oda H, Beker L, Kaizawa Y *et al.* A novel technology for free flap monitoring: pilot study of a wireless, biodegradable sensor. *J Reconstr Microsurg* 2020; **36**: 182-90.
3. Chao AH, Joseph M, P. PS *et al.* A review of devices used in the monitoring of microvascular free tissue transfers. *Expert Rev Med Devices* 2013; **10**: 649-60.
4. Chao AH, Lamp S. Current approaches to free flap monitoring. *Plast Aesthet Nurs* 2014; **34**: 57-8.
5. Kodama H, Ishida K, Hirayama H *et al.* The future of free flap monitoring by laser continuous doppler flowmetry: A prospective assessment in consecutive 71 patients. *JPRAS Open* 2025; **43**: 140-52.
6. Galla TJ, Hellekes D, Feller AM. Differentiation between arterial and venous vessel occlusion by simultaneous measurement with laser Doppler flowmetry and photoplethysmography. *J Reconstr Microsurg* 1999; **15**: 67-72.
7. Holzle F, Rau A, Loeffelbein DJ *et al.* Results of monitoring fasciocutaneous, myocutaneous, osteocutaneous and perforator flaps: 4-year experience with 166 cases. *Int J Oral Maxillofac Surg* 2010; **39**: 21-8.
8. Lacey H, Kanakopoulos D, Hussein S *et al.* Adjunctive technologies in postoperative free-flap monitoring: a systematic review. *J Plast Reconstr Aesthet Surg* 2023; **87**: 147-55.
9. Kagaya Y, Miyamoto S. A systematic review of near-infrared spectroscopy in flap monitoring: Current basic and clinical evidence and prospects. *J Plast Reconstr Aesthet Surg* 2018; **71**: 246-57.
10. Park JJ, Hong S, Jung Y *et al*. Highly sensitive cuffless blood pressure monitoring with selective laser-sintered liquid metal conductors. *Adv Funct Mater* 2025; **35**: 2505089.
11. Jiang Y, Trotsyuk AA, Niu S *et al.* Wireless, closed-loop, smart bandage with integrated sensors and stimulators for advanced wound care and accelerated healing. *Nat Biotechnol* 2023; **41**: 652-62.
12. Yang G, Zhu K, Guo W *et al.* Adhesive and hydrophobic bilayer hydrogel enabled on-skin biosensors for high-fidelity classification of human emotion. *Adv Funct Mater* 2022; **32**: 2200457.
13. Zhang L, Chen L, Wang S *et al.* Cellulose nanofiber-mediated manifold dynamic synergy enabling adhesive and photo-detachable hydrogel for self-powered E-skin. *Nat Commun* 2024; **15**: 3859.
14. Ma Z, Bourquard C, Gao Q *et al.* Controlled tough bioadhesion mediated by ultrasound. *Science* 2022; **377**: 751-5.
15. Chen X, Yuk H, Wu J *et al.* Instant tough bioadhesive with triggerable benign detachment. *PNAS* 2020; **117**: 15497-503.
16. Yang G, Lan Z, Gong H *et al.* A nepenthes-inspired hydrogel hybrid system for

sweat-wicking electrophysiological signal recording during exercises. *Adv Funct Mater* 2025; **35**: 2417841.
17. Gao Y, Wu K, Suo Z. Photodetachable adhesion. *Adv Mater* 2019; **31**: 1806948.
18. Borden LK, Gargava A, Raghavan SR. Reversible electroadhesion of hydrogels to animal tissues for suture-less repair of cuts or tears. *Nat Commun* 2021; **12**: 4419.
19. Yang G, Hu Y, Guo W *et al.* Tunable hydrogel electronics for diagnosis of peripheral neuropathy. *Adv Mater* 2024; **36**: 2308831.
20. Xue Y, Zhang J, Chen X *et al.* Trigger-detachable hydrogel adhesives for bioelectronic interfaces. *Adv Funct Mater* 2021; **31**: 2106446.
21. Xiao A, Jiang X, Hu Y *et al.* A degradable bioelectronic scaffold for localized cell transfection toward enhancing wound healing in a 3D space. *Adv Mater* 2024; **36**: 2404534.
22. Yang G, Qiu Y, Pang B *et al.* A reusable hydrogel biosensor array with electrically responsive hydrogel interfaces for noninvasive locating of perforating arteries. *Sci Adv* 2025; **11**: eadw6166.
23. Franklin D, Tzavelis A, Lee JY *et al.* Synchronized wearables for the detection of haemodynamic states via electrocardiography and multispectral photoplethysmography. *Nat Biomed Eng* 2023; **7**: 1229–41.
24. Wu H, Li Z, Xu Z *et al.* On-skin biosensors for noninvasive monitoring of postoperative free flaps and replanted digits. *Sci Transl Med* 2023; **15**: eabq1634.
25. Zhong Y, Zhang Y, Pu J *et al.* Monitoring blood pressure through a single hybrid hemodynamic signal with a flexible optoelectronic patch. *Device* 2025; **3**: 100778.
26. Lee HS, Noh B, Kong SU *et al.* Fiber-based quantum-dot pulse oximetry for wearable health monitoring with high wavelength selectivity and photoplethysmogram sensitivity. *npj Flex Electron* 2023; **7**: 15.
27. Wu W, Li L, Li Z *et al.* Extensible integrated system for real-time monitoring of cardiovascular physiological signals and limb health. *Adv Mater* 2023; **35**: 2304596.
